# Oligomerization of a plant helper NLR requires cell-surface and intracellular immune receptor activation

**DOI:** 10.1073/pnas.2210406120

**Published:** 2023-03-06

**Authors:** Joanna M. Feehan, Junli Wang, Xinhua Sun, Jihyeon Choi, Hee-Kyung Ahn, Bruno Pok Man Ngou, Jane E. Parker, Jonathan D. G. Jones

**Affiliations:** ^a^The Sainsbury Laboratory, University of East Anglia, Norwich NR4 7UH, UK; ^b^Department of Plant-Microbe interactions, Max Planck Institute for Plant Breeding Research, Cologne 50829, Germany

**Keywords:** plant immunity, NLR immune receptor proteins, TIR domain, defense signaling

## Abstract

Plant disease resistance involves both detection of microbial molecular patterns by cell-surface pattern recognition receptors and detection of pathogen effectors by intracellular NLR immune receptors. NLRs are classified as sensor NLRs, involved in effector detection, or helper NLRs required for sensor NLR signaling. TIR-domain-containing sensor NLRs (TNLs) require helper NLRs NRG1 and ADR1 for resistance, and helper NLR activation of defense requires the lipase-domain proteins EDS1, SAG101, and PAD4. Previously, we found that NRG1 associates with EDS1 and SAG101 in a TNL activation-dependent manner [X. Sun *et al.*, *Nat. Commun.* **12**, 3335 (2021)]. We report here how the helper NLR NRG1 associates with itself and with EDS1 and SAG101 during TNL-initiated immunity. Full immunity requires coactivation and mutual potentiation of cell-surface and intracellular immune receptor-initiated signaling [B. P. M. Ngou, H.-K. Ahn, P. Ding, J. D. G. Jones, *Nature* **592**, 110–115 (2021), M. Yuan *et al.*, *Nature* **592**, 105–109 (2021)]. We find that while activation of TNLs is sufficient to promote NRG1–EDS1–SAG101 interaction, the formation of an oligomeric NRG1–EDS1–SAG101 resistosome requires the additional coactivation of cell-surface receptor-initiated defense. These data suggest that NRG1–EDS1–SAG101 resistosome formation in vivo is part of the mechanism that links intracellular and cell-surface receptor signaling pathways.

## Significance

Plant immunity involves defense activation upon pathogen perception by both cell-surface and intracellular NLR immune receptors. Most plant NLRs detect and respond to pathogen effectors, whereas “helper” NLRs transduce signals initiated by “sensor” NLRs. We report here that pathogen detection by both cell-surface and intracellular immune receptors is required for activation of NRG1, a helper NLR essential for signaling from a subset of sensor NLRs. NRG1 is a broadly conserved helper NLR that activates defense via EDS1 and SAG101 lipase-like immune signaling components. Here, we reveal a recognition-dependent oligomeric NRG1–EDS1–SAG101 “resistosome” involved in plant immune receptor signaling.

Plants have powerful mechanisms that thwart attempted pathogen ingress and attenuate disease, but to be effective, they must be activated quickly. Rapid defense induction depends on pathogen recognition, which is achieved by both cell-surface and intracellular immune receptors that detect pathogen-derived molecules and initiate signaling that activates defense mechanisms (1).

Most plant disease *Resistance* (*R*) genes encode intracellular “NLR” immune receptors that carry a nucleotide-binding domain and a leucine-rich repeat domain. Many sensor NLRs that directly or indirectly detect pathogen effector molecules also require “helper” NLRs for their signaling (2). Most sensor NLRs carry either a TIR (Toll/Interleukin-1 receptor/Resistance) domain or a CC (coiled-coil) domain at their N-termini. All characterized TIR-NLRs (TNLs) require the helper NLR N-required gene 1 (NRG1) (and/or activated disease resistance gene 1 (ADR1) subfamilies for their full function (3). Structures of activated TNLs (4, 5) and CC-NLRs (CNLs) (6–8) reveal formation, respectively, of tetrameric or pentameric “resistosomes” upon immune activation. TNL resistosomes activate signaling via a TIR domain-dependent NADase activity (5), while hopZ-activated resistance 1 (ZAR1) resistosomes initiate defense signaling at the plasma membrane (PM) via cation channel formation (9).

The NRG1 and ADR1 subfamilies of helper NLRs and enhanced disease susceptibility 1 (EDS1)-family lipase-like proteins work together as two distinct nodes to mediate defense upon TNL-mediated effector recognition (10–15). In Arabidopsis, three genetically redundant ADR1s (ADR1, ADR1-L1, and ADR1-L2) cofunction with EDS1 and phytoalexin deficient 4 (PAD4) in potentiating transcriptional defenses which restrict pathogen growth, while NRG1s (NRG1.1 and NRG1.2) cofunction with EDS1 and senescence-associated gene 101 (SAG101) to execute a hypersensitive cell death response (HR). The NRG1 and ADR1 modules can partially substitute for the other in conferring pathogen resistance (10, 15). In *Nicotiana benthamiana*, the NRG1–EDS1–SAG101 module functions in both cell death and transcription, while the contribution of ADR1–EDS1–PAD4 remains unclear (12). Arabidopsis EDS1–PAD4 and EDS1–SAG101 dimers are receptors that bind distinct TIR enzyme-derived small molecules which allosterically induce their direct associations with ADR1 and NRG1 helper NLRs forming ADR1/EDS1/PAD4 or NRG1/EDS1/SAG101 complexes, respectively (16, 17). These data provide a biochemical mechanism for activation of the two EDS1—helper NLR modules, but it remains unknown how the induced complexes then function to activate transcriptional reprogramming, cell death, and defense.

Previously, we found that Arabidopsis NRG1 associates with EDS1 and SAG101 upon perception of effectors by TNL immune receptors in Arabidopsis (10). When transiently over-expressed in *N. benthamiana*, *At*NRG1.1 localizes to endomembrane networks (13, 18) while autoactive *At*NRG1A^D485V^ becomes localized to the PM to form a calcium-permeable cation channel potentially similar to ZAR1 (18). In contrast, Arabidopsis EDS1–SAG101 dimers primarily localize to the nucleus (12, 19). NRG1–EDS1–SAG101 can associate, eitherin nuclei or their periphery, in immune-activated tissue (10), and a recent report found small pools of EDS1, PAD4, ADR1, and SAG101 in close proximity to PM-localized SOBIR1 receptors (20). These reports illustrate the need for clear data that define the subcellular compartments(s) in which NRG1, EDS1, and SAG101 coimplement their immune functions upon defense activation.

Both cell-surface and intracellular immune receptor coactivation are required to mount full defense responses in plants (21, 22). Even though cell-surface and intracellular responses are mediated by distinct classes of immune receptors, they mutually potentiate shared pathways. ADR1 has been reported to play a role in cell-surface receptor responses (20, 23); however, no such role was reported for NRG1.

Using native promoter-driven stable Arabidopsis complementation lines, we investigated the assembly and subcellular localizations of NRG1–NRG1 and NRG1–EDS1–SAG101 interactions upon intracellular receptor-mediated and/or cell-surface receptor-initiated immune activation. Previously, we reported TNL activation-dependent NRG1 association with EDS1 and SAG101 (10). Here, we show that upon effector recognition by a TNL sensor, NRG1 is recruited into complexes with EDS1 and SAG101 that are detectable in the nucleus and at a location we term “PM/cytoplasm” (to indicate that our data are consistent with both locations). We find that NRG1–NRG1 association requires both cell-surface–initiated and intracellular receptor–mediated activation. In contrast, TNL activation alone can strongly induce NRG1 interaction with EDS1 and SAG101 and is sufficient for detection of heterotrimeric complexes. A slow-migrating NRG1–EDS1–SAG101 complex that is consistent with resistosome formation, into which only a small proportion of NRG1 protomers were incorporated, was only detected upon coactivation of intracellular and cell-surface receptor-mediated pathways. These data reveal a previously unknown requirement for coactivation of cell-surface and intracellular receptor signaling pathways in the formation of an NRG1-containing resistosome in vivo. This requirement has not been reported for other NLR resistosome assemblies. These data suggest NRG1–EDS1–SAG101 interactions are part of the mechanisms that link cell-surface and intracellular receptor-mediated defense responses in plants.

## Results

### NRG1 Associates with EDS1 and SAG101 at the Plasma Membrane and Nucleus upon Effector-Dependent Defense Activation.

We previously reported that NRG1 associates with EDS1 and SAG101 upon TNL-effector recognition in Arabidopsis (10). To investigate further the role of these components in TNL-mediated immune signaling, we generated stable transgenic lines in a Col-0 *nrg1.1 nrg1.2 sag101-3* mutant background (10) carrying complementing pNRG1.2:NRG1.2-FLAG, pSAG101:SAG101-HA, and pEDS1:EDS1-V5 (*SI Appendix*, Fig. S1 *A* and *B*). As previously (10), we utilized the Pf0-1 (*Pseudomonas fluorescens*) EtHAn (Effector-to-Host Analyzer) system (24) (hereafter referred to as “Pf0-1”) for delivery of effector AvrRps4 that is recognized by the endogenous Arabidopsis TNL pairs RRS1/RPS4 and RRS1B/RPS4B (25) to test induction of NRG1 association with EDS1 and SAG101.

Leaves of transgenic Arabidopsis carrying epitope-tagged NRG1, EDS1, and SAG101 were infiltrated with Pf0-1 carrying either AvrRps4 or nonrecognized AvrRps4^EEAA^ (26) or were mock-infiltrated with MgCl_2_ before harvest at 8 hours postinfiltration (hpi) and immunoprecipitation (IP). Noninfiltrated leaves were harvested as preactivation control. We observed a previously unnoticed weak, constitutive EDS1-V5 and SAG101-HA association with NRG1.2-FLAG (*SI Appendix*, Fig. S1*A*). We infer that the weak association in these Arabidopsis lines is detected due to a higher sensitivity of α-HA and α-V5 epitope tags compared to earlier studies (10). Both Pf0-1 AvrRps4^EEAA^- and MgCl_2_-infiltrated leaves showed a slight enhancement of EDS1-V5 and SAG101-HA association with NRG1.2-FLAG compared to uninfiltrated control (*SI Appendix*, Fig. S1*A*). These data show that cell-surface receptor activation, initiated either by pathogen-associated or mock infiltration–induced damage, weakly induces EDS1 and SAG101 association with NRG1. While this was not previously observed in Arabidopsis assays, transient *N. benthamiana* Agro-infiltration assays, which likely activate cell-surface receptors, triggered a weak NRG1 association with EDS1 and SAG101 (10). We verified our previous finding that EDS1-V5 and SAG101-HA association with NRG1.2-FLAG is strongly enhanced upon AvrRps4 effector delivery (10) (*SI Appendix*, Fig. S1*A*). The early NRG1 interaction with EDS1 and SAG101 (4 hpi), which accumulates over time (8 hpi) as observed in transient *N. benthamiana assays* (10), was also detected here in stable Arabidopsis lines (*SI Appendix*, Fig. S1*A*). Collectively, we infer that there may be a dynamic on–off equilibrium of NRG1 with EDS1 and SAG101 that is stabilized by TNL-generated small molecules upon effector activation (17).

**Fig. 1.**
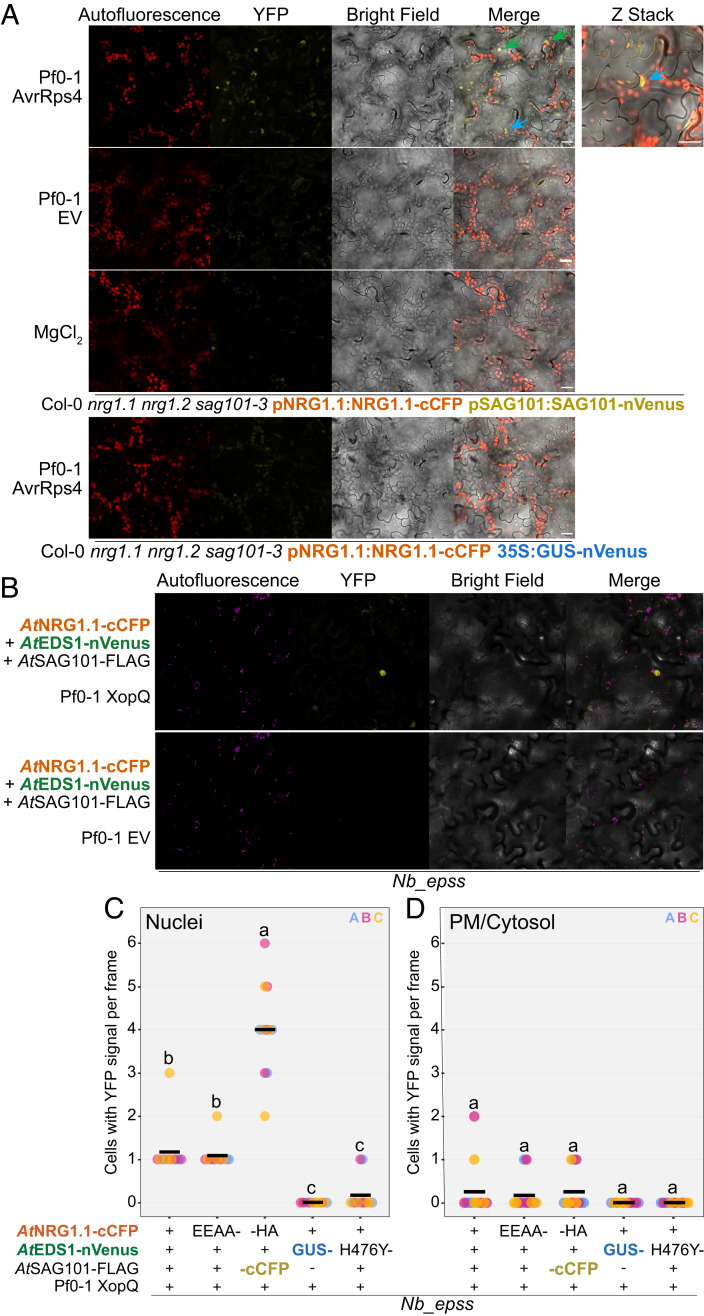
NRG1/EDS1 and NRG1/SAG101 associate in nuclei and at the PM/cytoplasm. (*A*) NRG1 associates with SAG101 in nuclei and at the PM/cytoplasm post Pf0-1 AvrRps4 delivery in Arabidopsis. Live cell imaging of native promoter-driven Arabidopsis stable lines for BiFC assays. Micrographs were taken 4 to 5 hpi of Pf0-1 and all images show single planes, excluding Z-stack image. Three biological replicates were performed with similar results, and representative micrographs are shown. (Scale bar, 10 μm.) Arrows indicate nuclei. Blue arrows indicate same nucleus between images. Autofluorescence (red) and YFP (yellow) signal are shown. (*B*) NRG1 associates with EDS1 in nuclei and at the PM/cytoplasm post Pf0-1 XopQ delivery in *N. benthamiana*. *Agrobacteria* carrying 35S promoter-driven BiFC constructs were infiltrated into *Nb_epss* leaves. At 48 hpi, Pf0-1 was infiltrated. At 4 to 6 hpi, leaf disks were imaged. Representative images are shown for four independent biological replicates with similar results. Autofluorescence (magenta) and YFP (yellow) signal are shown. (*C*) More cells have detectable YFP signal in the nucleus when NRG1 or NRG1^EEAA^ is codelivered with EDS1, than when NRG1 is codelivered with GUS or EDS1^H476Y^. Numbers of cells with BiFC-mediated YFP signal in the nucleus in activated *N. benthamiana* leaves were counted. Experiments were performed three times independently, each with four leaf-disc replicates (n = 12). Mean indicated at black line. ANOVA, *P* < 0.001, Tukey HSD, *P* < 0.05. (*D*) YFP signal at the PM/cytosol is not statistically different when NRG1 is codelivered with EDS1 compared to negative controls. Numbers of cells with BiFC-mediated YFP signal in PM/cytoplasm in activated *N. benthamiana* leaves were counted. Experiments were performed three times independently, each with four leaf-disc replicates (n = 12). Mean indicated at black line. ANOVA not statistically different; however, at least one technical replicate in two of three biological replicates had signal with NRG1/EDS1 and NRG1^EEAA^/EDS1 like the EDS1/SAG101 positive control. "EEAA-" refers to *At*NRG1.1^EEAA^-cCFP, "-HA" refers to *At*NRG1.1-HA, "GUS-" refers to GUS-nVenus, "H476Y-" refers to *At*EDS1^H476Y^-nVenus, "-cCFP" refers to *At*SAG101-cCFP, "-FLAG" refers to *At*EDS1-FLAG, and "-nVenus" refers to *At*SAG101-nVenus. Data generated with ggplot2 (3.3.2) package in R.

We investigated the subcellular locations of the induced EDS1 and SAG101 complexes with NRG1 upon effector recognition utilizing bimolecular fluorescence complementation (BiFC) assays. Detection of yellow fluorescent protein (YFP) signals in BiFC assays indicates components are within sufficient proximity to reconstitute fluorophore excitation, revealing their current location but not necessarily where the complex was formed. We complemented Col-0 *nrg1.1 nrg1.2 sag101-3* with pNRG1.1:NRG1.1-cCFP and pSAG101:SAG101-nVenus (*SI Appendix*, Fig. S1*C*) for NRG1–SAG101 BiFC assays. NRG1–EDS1 BiFC assays were carried out via Agro-mediated transient expression of 35S promoter-driven *At*NRG1.1-cCFP with *At*EDS1-nVenus and *At*SAG101-Flag in *N. benthamiana*, which are functional in reconstituting cell death (*SI Appendix*, Fig. S2*A*). An enhanced YFP signal in nuclei and PM/cytoplasm was detected upon Pf0-1 AvrRps4 delivery in Arabidopsis (Fig. 1*A*) and upon Pf0-1 XopQ delivery—recognized by the TNL Roq1—in *N. benthamiana* (Fig. 1*B*). We confirmed in activated *N. benthamiana* tissue that a YFP signal is detectable only when *At*NRG1.1-cCFP and *At*EDS1-nVenus are codelivered (*SI Appendix*, Fig. S2*D*). In the absence of an effector, the YFP signal produced by *At*NRG1/*At*EDS1 and *At*NRG1/*At*SAG101 BiFC constructs was not detected (*SI Appendix*, Fig. S2 *B* and *C*). These data indicate that effector-dependent NRG1–SAG101 and NRG1–EDS1 associations are present at multiple locations within the cell, including previously undetected NRG1–EDS1–SAG101 complexes inside the nucleus.

To further validate the signal at each subcellular location, we used as negative controls an *At*NRG1^EEAA^ mutant that maintains association with *At*EDS1 and *At*SAG101 but does not elicit TNL-activated cell death, and an *At*EDS1^H476Y^ mutant that neither associates with *At*NRG1 nor elicits cell death (10). We counted the number of cells with the YFP signal in nuclei or PM/cytoplasm in TNL-activated *N. benthamiana* leaves. More nuclei showed YFP signal when tagged *At*NRG1/*At*EDS1 or *At*NRG1^EEAA^/*At*EDS1 were codelivered compared to the negative controls *At*EDS1^H476Y^ or GUS (Fig. 1*C*). These data support the presence of a nuclear-localized NRG1–EDS1–SAG101 complex.

Notably, there was no significant difference between samples in the number of cells with YFP signal at the PM/cytoplasm (Fig. 1*D*). However, signal was detected in one of four technical replicates in two of the three biological replicates upon codelivery of tagged *At*NRG1 or *At*NRG1^EEAA^ with *At*EDS1, while no signal was ever detected for *At*NRG1 together with the negative controls *At*EDS1^H476Y^ or GUS (Fig. 1*D*). Importantly, the number of cells with *At*NRG1/*At*EDS1 or *At*NRG1^EEAA^/*At*EDS1 signal at the PM/cytoplasm was indistinguishable from the number in the *At*EDS1/*At*SAG101 positive control (Fig. 1*C*). This low signal in PM/cytoplasm may be explained by a low abundance of *At*SAG101 outside the nucleus (12, 19).

No data were previously reported that unambiguously support a nuclear-localized mechanism for NRG1 in defense responses (10, 13, 18). Therefore, we independently verified nuclear localization of NRG1 utilizing Pf0-1 delivery of AvrRps4 in Col-0 *nrg1.1 nrg1.2* carrying pNRG1.1:NRG1.1-mRuby (*SI Appendix*, Fig. S3*A*). Upon Pf0-1 delivery of AvrRps4, an increased mRuby signal was detected in nuclei (*SI Appendix*, Fig. S3*C*). Although a greater overall accumulation of NRG1 was also detected upon AvrRps4 delivery (*SI Appendix*, Fig. S3*B*), these data indicate that NRG1 may either relocalize to nuclei upon effector recognition or is present in low abundance prior to immune activation. Importantly, they support our observations of a previously unreported NRG1 association with EDS1 and SAG101 in the nucleus.

### NRG1 Oligomerizes upon Effector-Dependent Defense Activation in Arabidopsis.

**Fig. 2.**
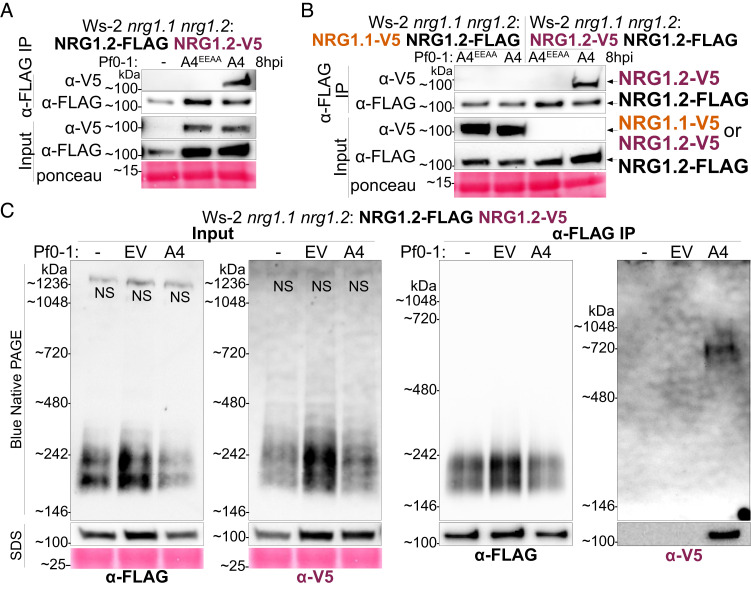
NRG1 self-associates upon effector recognition in Arabidopsis. (*A*) NRG1.2-FLAG associates with NRG1.2-V5 upon AvrRps4 delivery in Arabidopsis. (*B*) NRG1 self-association is paralog specific. In (*A*) and (*B*): SDS-PAGE and western blots of coIPs performed with native promoter-driven Arabidopsis stable lines. Experiments were performed on three biological replicates each for two independent lines, for a total of six replicates, with similar results. While input for NRG1.2-V5 not detected in (*B*) α-FLAG IP shows NRG1.2-V5 coIPs with NRG1.2-FLAG as the positive control. (*C*) Effector-dependent formation of an NRG1 oligomer in Arabidopsis. BN-PAGE and western blot of lysate and coIP elution products performed with native promoter-driven Arabidopsis stable line. Experiments were performed on three biological replicates with similar results. "NS" indicates nonspecific band. "A4" indicates AvrRps4, "A4^EEAA^" indicates AvrRps4^EEAA^, "EV" indicates empty vector, and "-" indicates uninfiltrated. NativeMark™ Unstained Protein Standard used in (*C*).

We investigated self-association of NRG1 in Arabidopsis before and after immune activation in supertransformed lines of Ws-2 *nrg1.1 nrg1.2* pNRG1.2:NRG1.2-FLAG (14) carrying an additional epitope-tagged NRG1, pNRG1.2:NRG1.2-V5. Self-association of NRG1 was evaluated by α-FLAG IP of NRG1.2-FLAG followed by α-V5 immunodetection of NRG1.2-V5. We tested this association after Pf0-1 delivery of AvrRps4 or AvrRps4^EEAA^, with uninfiltrated leaves as a preactivation control. We found that NRG1.2-V5 associated with NRG1.2-FLAG only after AvrRps4 delivery (Fig. 2*A*). We attempted to determine the subcellular location of self-associating NRG1. However, we could not detect an unambiguous YFP signal in *N. benthamiana* leaves upon codelivery of *At*NRG1.1-cCFP and *At*NRG1.1-nVenus with Pf0-1 delivery of XopQ at 48 hpi (*SI Appendix,* Fig. S4). We conclude that NRG1 does not interact with itself in the preactivation state, or after surface receptor activation, but only upon coactivation of cell-surface and TNL immune receptors. The subcellular location of oligomerized NRG1 remains to be determined.

We also investigated whether NRG1 self-association is paralog specific. We super-transformed the previously generated Ws-2 *nrg1.1 nrg1.2* pNRG1.1:NRG1.1-V5 (14) to carry an additional variant of pNRG1.2:NRG1.2-FLAG. Unlike NRG1.2-V5, NRG1.1-V5 did not associate with NRG1.2-FLAG upon delivery of AvrRps4 (Fig. 2*B*). The input signal for NRG1.2-V5 was not detected when imaged in parallel with NRG1.1-V5, indicating lower accumulation of NRG1.2 compared to NRG1.1 in Arabidopsis. However, the positive control coimmunoprecipitation (coIP) of NRG1.2-V5 after α-FLAG IP of NRG1.2-FLAG shows that NRG1.2-V5 is expressed in this line. NRG1.2-V5 was also detected in the input samples of Fig. 2 *A* and *C*. These data highlight the paralog specificity of NRG1–NRG1 self-association.

Expression of autoactive *At*NRG1.1^D485V^ in *N. benthamiana* revealed slower migrating forms in Blue Native polyacrylamide gel electrophoresis (BN-PAGE) assays (18) that may indicate NRG1 oligomers. To investigate NRG1 oligomer formation in Arabidopsis, we subjected lysates and coIP samples from Ws-2 *nrg1.1 nrg1.2* pNRG1.2:NRG1.2-FLAG pNRG1.2:NRG1.2-V5 to BN-PAGE assays (see *Materials and Methods* for BN-PAGE marker details). Species of NRG1.2-FLAG or NRG1.2-V5 were immunolabelled after α-FLAG IP of NRG1.2-FLAG. Slower migrating forms of NRG1.2-FLAG and NRG1.2-V5 were not observed in lysates after Pf0-1 AvrRps4 delivery in Arabidopsis. However, a slower migrating form of NRG1.2-V5 was observed in Pf0-1 AvrRps4-treated tissues after α-FLAG IP of NRG1.2-FLAG (Fig. 2*C*). Notably, no slow-migrating NRG1.2-FLAG was detected after α-FLAG IP of NRG1.2-FLAG in Pf0-1 AvrRps4-treated tissue, even with overexposure (*SI Appendix*, Fig. S5). These data likely indicate that in Arabidopsis, AvrRps4 delivery and TNL activation result in a small proportion of total NRG1 protein being converted into an NRG1 oligomer.

### Effector-Dependent Association of EDS1 and SAG101 with an NRG1 Oligomer in Arabidopsis.

**Fig. 3.**
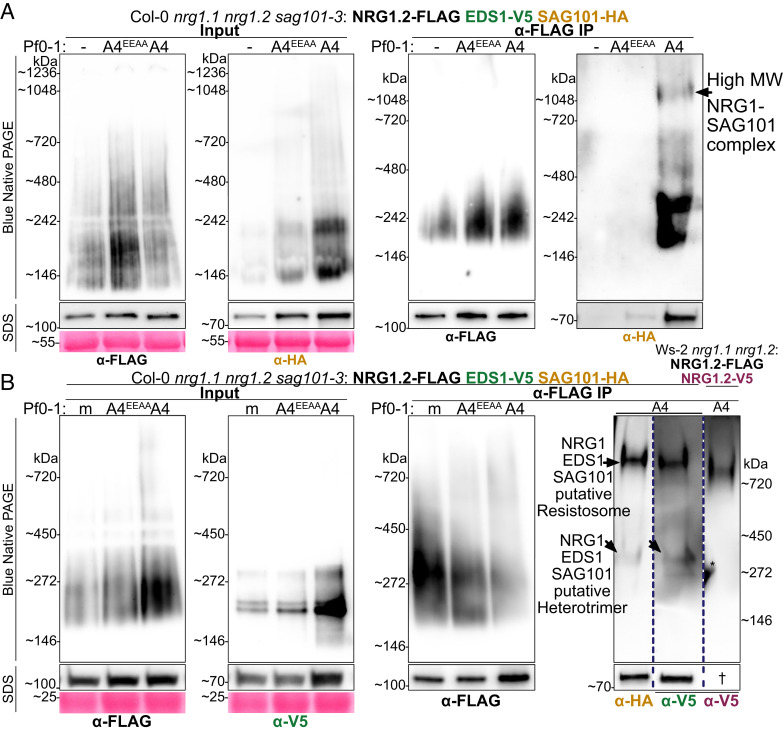
Formation of NRG1 oligomer in complex with EDS1 and SAG101 requires effector recognition in Arabidopsis. BN-PAGE and western blot of lysates and coIP elution products performed with native promoter-driven Arabidopsis stable lines. Tissue was harvested 8 hpi. Experiments were performed on at least three biological replicates with similar results. (*A*) Higher-order forms of NRG1–SAG101 complexes require effector delivery in Arabidopsis. (*B*) NRG1 oligomer comigrates with the higher-order form of NRG1-associated EDS1–SAG101 in Arabidopsis after effector delivery. Blue dotted line in BN-PAGE indicates samples were resolved on the same gel and western blot images were cropped together. Blue dotted line in SDS-PAGE indicates lanes were cropped from western blot images of samples resolved in separate gels. "A4" indicates AvrRps4, "A4^EEAA^" indicates AvrRps4^EEAA^, "-" indicates un-infiltrated, and "m" is MgCl_2_ mock infiltration. Asterisk (*) indicates signal is bleed through from adjacent lane not shown. Dagger “†” indicates image shown in input panel of Fig. 2*B*. NativeMark™ Unstained Protein Standard used in (*A*) while SERVA marker used in (*B*).

We investigated whether EDS1 and SAG101 interact with the NRG1 oligomer in an NRG1–EDS1–SAG101 resistosome-like complex. We subjected pre- and post-activation lysates and coIP samples from Col-0 *nrg1.1 nrg1.2 sag101-3* pNRG1.2:NRG1.2-FLAG pSAG101:SAG101-HA pEDS1:EDS1-V5 to BN-PAGE assays. As with NRG1.2 (Fig. 2*C*), slower migrating forms of SAG101-HA and EDS1-V5 were not observed in lysates after Pf0-1 AvrRps4 delivery in Arabidopsis (Fig. 3 *A* and *B*). However, multiple forms of SAG101-HA were detected in BN-PAGE after α-FLAG IP of NRG1.2-FLAG in Pf0-1 AvrRps4-treated tissues, including a slow-migrating species above the 720-kDa marker (Fig. 3*A*). This contrasts with the presence of a single NRG1.2-V5 species after α-FLAG IP of NRG1.2-FLAG (Fig. 2*C*). Although SAG101-HA and EDS1-V5 weakly associated with NRG1.2-FLAG in preactivation states (*SI Appendix*, Fig. S1*A*), no SAG101-HA signal was detected after α-FLAG IP of NRG1.2-FLAG in uninfiltrated or Pf0-1 AvrRps4^EEAA^ infiltrated tissue (Fig. 3*A*), suggesting any preactivation associations are of too low abundance to be detected. These data indicate the formation of multiple NRG1–EDS1–SAG101 complexes, rather than a single detectable species of an NRG1–NRG1 oligomer, upon immune activation in Arabidopsis.

Heterodimers of EDS1–SAG101 were previously found to elute near the 158-kDa marker in size exclusion chromatography (19) and a putative EDS1–SAG101 heterodimer migrates between 146- and 242-kDa markers in BN-PAGE assays (*SI Appendix*, Fig. S6). A species of SAG101-HA and EDS1-V5 was detected after α-FLAG IP of NRG1.2-FLAG in AvrRps4-treated tissues migrating just above the ~272-kDa marker (Fig. 3*B*). It is possible these data represent degradation of NRG1–EDS1–SAG101 complexes into NRG1 monomers and EDS1–SAG101 heterodimers. However, an effector-dependent, low molecular weight species of SAG101-HA still migrated slower than the preactivation species of SAG101-HA, EDS1-V5, or NRG1.2-FLAG (*SI Appendix*, Fig. S6). This activated complex contains NRG1.2-FLAG as it was isolated by α-FLAG IP. However, an NRG1.2-FLAG species is not detected near the ~272-kDa marker and only the oligomeric species is detected above the ~720-kDa marker (Figs. 2*C* and 3*B*). We interpret these collective data to reflect formation of a putative NRG1–EDS1–SAG101 heterotrimer (NRG1 monomer in complex with an EDS1–SAG101 heterodimer) upon effector recognition in Arabidopsis.

To investigate whether the slower migrating forms of NRG1, SAG101, and EDS1 correspond to the same molecular complex, elution products after α-FLAG IP of NRG1.2-FLAG from either Col-0 *nrg1.1 nrg1.2 sag101-3* pNRG1.2:NRG1.2-FLAG pSAG101:SAG101-HA pEDS1:EDS1-V5 or Ws-2 *nrg1.1 nrg1.2* pNRG1.2:NRG1.2-FLAG pNRG1.2:NRG1.2-V5 were resolved by BN-PAGE in parallel. Immunolabelling with α-HA for SAG101-HA and α-V5 for EDS1-V5 or NRG1.2-V5 revealed that AvrRps4-induced slow migrating forms above 720 kDa of SAG101-HA, EDS1-V5, and NRG1.2-V5 after α-FLAG IP of NRG1.2-FLAG migrate at an indistinguishable size—consistent with an NRG1 oligomer in complex with EDS1 and SAG101 (Fig. 3*B* and *SI Appendix*, Fig. S7). These data indicate that effector recognition in Arabidopsis leads to accumulation of a putative NRG1–EDS1–SAG101 resistosome.

### Effector Recognition Is Sufficient to Induce NRG1/EDS1 and NRG1/SAG101 Association and Requires TIR-Domain Enzyme Activity.

Plant TIR domains form holoenzymes which can produce a suite of cyclic and noncyclic small molecules using NAD+ as an initial substrate (3–5, 16, 17, 27). Products include nicotinamide and several ADPR-like molecules such as di-ADPR or ADPR-ATP that activate EDS1-dependent defense but which are insufficient for cell death (17, 27–30). We investigated whether TIR enzymatic activity is required for EDS1 and SAG101 association with NRG1 using Agro-infiltration in *N. benthamiana.* Expression of TIR-only Arabidopsis Response to the bacterial type III effector protein HopBA1 (RBA1) is sufficient for ADR1-induced association with EDS1-family proteins in *N. benthamiana* (31). Utilizing a cell death inactive NRG1.2^EEAA^ mutant (*SI Appendix*, Fig. S8) which maintains TNL-induced association with EDS1 and SAG101 (10), we tested whether NRG1 could associate with EDS1 and SAG101 in the presence of RBA1 or NADase mutant RBA1^E86A^. We found that EDS1-V5 and SAG101-Myc associate with NRG1.2^EEAA^-FLAG when coexpressed with RBA1 but not with enzymatically inactive RBA1^E86A^ (*SI Appendix*, Fig. S9). These data indicate that TIR domain enzymatic activity is required for EDS1 and SAG101 association with NRG1 in vivo.

**Fig. 4.**
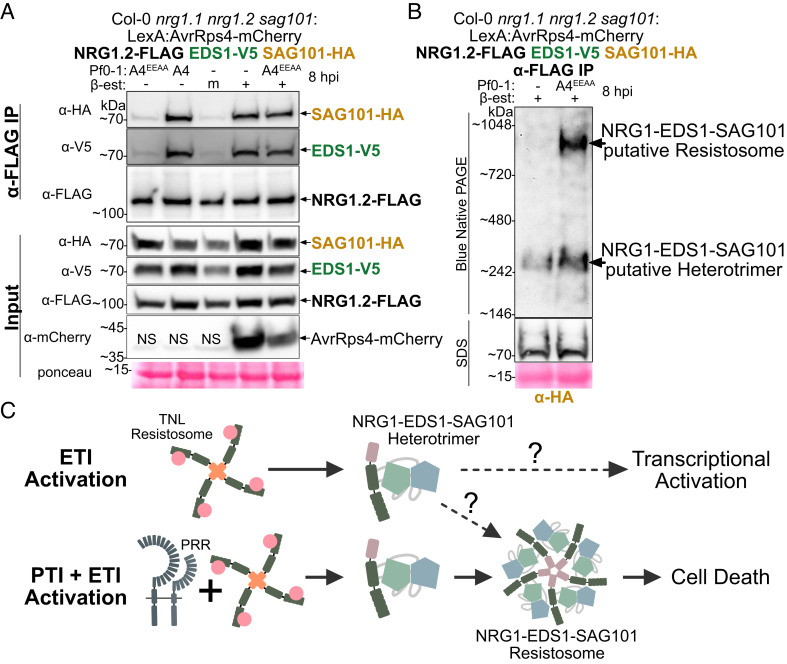
Both PTI and ETI activation are required for accumulation of NRG1–EDS1–SAG101 oligomeric "resistosome" like complex. (*A*) ETI alone is sufficient to induce NRG1/EDS1 and NRG1/SAG101 associations in Arabidopsis. SDS-PAGE and western blot of lysates and coIP elution products from native promoter-driven Arabidopsis stable lines. Experiments were performed on three biological replicates each for two independent lines, for a total of six replicates, with similar results. (*B*) Both ETI and PTI activation are required for accumulation of an NRG1–EDS1–SAG101 putative resistosome. BN-PAGE and western blot of coIP elution products from native promoter-driven Arabidopsis stable lines. Experiments were performed on three biological replicates with similar results. NativeMark™ Unstained Protein Standard was used. In (*A*) and (*B*), "m" indicates mock infiltration, "NS" indicates nonspecific band, and "-" indicates no infiltration. (*C*) Proposed activation and oligomerization of NRG1 with EDS1 and SAG101. Upon TNL binding of effector ligands, ETI is activated and NRG1 is induced to associate with EDS1 and SAG101 to form an NRG1–EDS1–SAG101 heterotrimer, which possibly induces transcriptional activation in nuclei. When PTI and ETI are coactivated, both NRG1–EDS1–SAG101 heterotrimers and resistosomes are formed, the latter of which possibly facilitates cell death via Ca^2+^ channel formation at the PM. It is not yet clear if heterotrimer formation is required prior to oligomerization and resistosome formation. "ETI" stands for effector-triggered immunity. "PTI" refers to pattern-triggered immunity. Dotted lines indicate hypothetical pathways that require further investigation.

Macroscopic cell death in Arabidopsis, indicated by tissue collapse, requires both cell-surface receptor activation (PAMP-triggered immunity or “PTI”) and effector recognition by intracellular receptors (effector-triggered immunity or “ETI”) (21, 22). We investigated whether ETI in the absence of PTI is sufficient for NRG1 association with EDS1 and SAG101. We generated a Col-0 *nrg1.1 nrg1.2 sag101-3* line carrying pNRG1.2:NRG1.2-FLAG pSAG101:SAG101-HA pEDS1:EDS1-V5 with a β-estradiol-inducible AvrRps4-mCherry driven by the LexA promoter with the XVE system (32) (*SI Appendix*, Fig. S10*A*). Leaves were infiltrated with β-estradiol to induce AvrRps4-mCherry expression to activate ETI alone or coinfiltrated with β-estradiol and Pf0-1 AvrRps4^EEAA^ for reconstitution of PTI+ETI coactivation. As previously, β-estradiol induction of AvrRps4 (ETI) is sufficient for defense gene activation (21) and induces weak microscopic cell death without tissue collapse, while β-estradiol induction of AvrRps4-mCherry in the presence of Pf0-1 AvrRps4^EEAA^ (PTI + ETI reconstitution) induces strong macroscopic cell death phenocopying Pf0-1 AvrRps4 delivery (PTI + ETI) (33) (*SI Appendix*, Fig. S10). We found that induction of AvrRps4-mCherry by β-estradiol infiltration is sufficient to induce EDS1-V5 and SAG101-HA association with NRG1.2-FLAG (Fig. 4*A*). Notably, the SAG101-HA and EDS1-V5 signal after α-FLAG IP of NRG1.2-FLAG was indistinguishable between β-estradiol (ETI), Pf0-1 AvrRps4 (PTI + ETI), and β-estradiol with Pf0-1 AvrRps4^EEAA^ (PTI + ETI reconstitution). These data indicate that ETI alone is sufficient to induce EDS1 and SAG101 interaction with NRG1 in Arabidopsis and to a level comparable with PTI and ETI coactivation.

### PTI and ETI Coactivation Promotes Accumulation of the NRG1–EDS1–SAG101 Oligomeric Complex.

To test whether β-estradiol-induction of NRG1–EDS1–SAG101 complexes is sufficient for the formation of NRG1–EDS1–SAG101 resistosomes, we performed BN-PAGE with coIP samples from Col-0 *nrg1.1 nrg1.2 sag101-3* carrying pNRG1.2:NRG1.2-FLAG pSAG101:SAG101-HA pEDS1:EDS1-V5 pLexA:AvrRps4-mCherry XVE after β-estradiol induction of AvrRps4-mCherry (ETI) or β-estradiol coinfiltration with Pf0-1 AvrRps4^EEAA^ (PTI + ETI reconstitution). As expected, β-estradiol coinfiltration with Pf0-1 AvrRps4^EEAA^ induced the accumulation of a slow-migrating SAG101-HA species, above the 720-kDa marker, after α-FLAG IP of NRG1.2-FLAG (Fig. 4*B*). However, this high molecular weight species was not detected after β-estradiol induction of AvrRps4-mCherry, and only the low molecular weight SAG101-HA species above the 242-kDa marker is present (Fig. 4*B*). Although ETI is sufficient for NRG1 interaction with EDS1 and SAG101 (Fig. 4*A*), these data indicate that ETI is not sufficient for the accumulation of high molecular weight NRG1–EDS1–SAG101 complexes. This finding highlights a requirement for an unknown PTI activation-dependent mechanism facilitating in vivo assembly of a putative NRG1–EDS1–SAG101 resistosome.

## Discussion

Coactivation and mutual potentiation of cell-surface receptor (PTI) and intracellular receptor (ETI) immune systems are required to mount a pathogen-restricting defense and host cell death response in Arabidopsis (21, 22), but the mechanism(s) by which PTI enhances cell death with ETI is unknown. We show here that while ETI alone is sufficient for NRG1 association with EDS1 and SAG101 (Fig. 4*A*), ETI is not sufficient for the formation of a stable NRG1–EDS1–SAG101 putative resistosome (Fig. 4*B*). Formation of higher-order NRG1 complexes was reported to correlate with activation of cell death (18). Our data suggest that PTI is required for TNL-dependent cell death because it potentiates formation of a high molecular weight NRG1–EDS1–SAG101 resistosome-like complex.

As the Arabidopsis NRG1.1 and NRG1.2 paralogs are functionally indistinguishable (13, 14), and as fusion tagging can perturb NRG1 function, specifically fluorophore-tagging of NRG1.2 (12), different paralogs were employed in our study to ensure that investigations were performed with functional tagged alleles (see *Materials and Methods*, NRG1.1 and NRG1.2). Previously, it was shown that both NRG1.1 and NRG1.2 associate with EDS1 and SAG101 upon effector recognition in *N. benthamiana* transient assays and Arabidopsis stable lines, respectively (10). These data indicate that NRG1.1 and NRG1.2 activation mechanisms are similar. We show that NRG1.2 self-association is paralog specific, yet it remains possible that NRG1.1 oligomerization could be distinct. It is, however, unlikely that NRG1.1 does not oligomerize upon activation as the autoactive NRG1.1^D485V^ was shown to migrate slower in BN-PAGE (18). Future studies could be designed to evaluate NRG1.1–NRG1.1 self-association upon effector delivery in Arabidopsis stable lines. Additionally, follow-up studies could confirm that NRG1.2 associations with EDS1 and SAG101 are detectable in nuclei and PM/cytoplasm, although this will require careful construction and functional validation of materials. Conceivably, NRG1.1 and NRG1.2 have distinct activation mechanisms, but we consider this unlikely.

The helper NLR NRG1 is essential for TNL-mediated cell death via a mechanism that involves EDS1 and SAG101 (10–15). A related helper NLR, ADR1, functions together with EDS1 and PAD4 for transcriptional reprogramming of defense genes (10, 15, 20, 23, 31, 34). These modules appear to only partially compensate for each other’s absence in Arabidopsis (10, 15). Therefore, we speculate that TNL-triggered cell death and transcription mediated by NRG1–EDS1–SAG101 could be carried out by distinct complexes that are spatially separated within the cell: resistosomes at the PM and potentially heterotrimers in the nucleus. We confirmed the requirement for PTI and ETI coactivation in macroscopic cell death (33) (*SI Appendix*, Fig. S10*A*) and found NRG1 associated with EDS1 and SAG101 at the PM/cytoplasm and in nuclei (Fig. 1 *A*–*D*). As the putative NRG1–EDS1–SAG101 resistosome is detected after PTI and ETI coactivation (Fig. 4*B*), we hypothesize that this form contributes to cell death at the PM. Indeed, previous reports identify a cell death mechanism for an oligomerized, constitutively active NRG1 at the PM (18). We also confirmed the sufficiency of ETI activation for transcription elevation of defense genes (21) (*SI Appendix*, Fig. S10*B*). The lack of a detectable NRG1–EDS1–SAG101 resistosome during ETI alone (Fig. 4*B*) points to the possibility of NRG1–EDS1–SAG101 heterotrimers mediating transcriptional defense responses in the nucleus. Our inference that NRG1 has a nuclear activity is consistent with earlier studies showing that nucleus-limited EDS1 restricts pathogen growth in Arabidopsis (35, 36). How heterotrimer immune complexes in nuclei, containing nonoligomerized helper NLRs, might function in transcriptional defense remains unclear and is not hypothesized for other NLRs, such as ZAR1 which oligomerizes to form plasma membrane Ca^2+^ channels (9). Our study highlights the possibility that oligomeric and heterotrimeric helper NLR forms may have distinct functions in mediating defense outputs in plants.

In contrast to previous studies which showed 100% conversion of NRG1.1 to slower-migrating species of autoactive NRG1.1^D485V^ in BN-PAGE (18), only a small proportion of NRG1.2, EDS1, and SAG101 protein was converted to putative resistosomes in Arabidopsis (Figs. 2*C* and 3). This also contrasts with the quantitative conversion of inactive to oligomerized helper NLR NRC in Solanaceae (37, 38). It may be that very few activated forms of NRG1–EDS1–SAG101 resistosomes are sufficient for defense by potentially increasing Ca^2+^ influx to the cytoplasm (13). However, low detection of high molecular weight complexes might represent localization changes that inhibit extraction or conformational changes that bury epitopes or result in steric hindrance. This may explain why a YFP signal in NRG1–NRG1 self-association BiFC assays could not be detected (*SI Appendix*, Fig. S4), or why so few NRG1–EDS1 and NRG1–SAG101 complexes were detected in PM/cytoplasm (Fig. 1). Low detection of resistosome accumulation could also be influenced by NRG1–EDS1–SAG101 oligomeric complex instability. Perhaps, EDS1–SAG101 promotes NRG1 oligomerization and then exits the complex, leaving a putative NRG1 pentamer. Indeed, faster migrating species of EDS1–SAG101 were detected after IP of NRG1 in activated tissues, including a species <242 kDa and lower molecular weight than the putative heterotrimer (Figs. 3 *A* and *B* and 4*B*). This could be interpreted as EDS1–SAG101 dissociation from NRG1 oligomers. However, only one stable form of oligomeric NRG1 was observed in vivo (Fig. 2*C*) which migrated indistinguishably from the high molecular weight species of SAG101 and EDS1 after IP of NRG1 in activated tissues (Fig. 3*B*). This indicates that once putative NRG1–EDS1–SAG101 resistosomes are formed, they are stable, resembling the ZAR1 pentamer which remains stably associated with RKS1/PBL2^ump^ in resistosomes once formed (6, 7). Our data indicate the stable formation of an immune activation-dependent putative NRG1–EDS1–SAG101 resistosome in Arabidopsis and highlight the importance of investigating NRG1 molecular assemblies and subcellular sites of action in the presence of EDS1 and SAG101.

The requirement for PTI and ETI coactivation in TNL-mediated cell death (33) could be explained by our observation that PTI and ETI coactivation is required for detection of putative NRG1–EDS1–SAG101 resistosomes (Fig. 4*B*). We show that TIR domain NADase activity is necessary for NRG1 stable association with EDS1 and SAG101 (*SI Appendix*, Fig. S9). Indeed, TNL-mediated ETI activation leads to interaction of NRG1–EDS1–SAG101 (17) and ADR1–EDS1–PAD4 (16) complexes via TIR domain-mediated biosynthesis of distinct small molecules derived from NAD+. Likely, the production and availability of distinct NAD-derived small molecules dictate the activation of NRG1–EDS1–SAG101 versus ADR1–EDS1–PAD4 signaling pathways. Small-molecule–induced NRG1–EDS1–SAG101 heterotrimer formation is analogous to formation of ZAR1–RKS1 intermediate species upon interaction with uridylylated PBL2 (6). Conceivably, steric changes induced by NRG1–EDS1–SAG101 heterotrimer formation enable resistosome assembly in a similar manner to ZAR1–RKS1–PBL2^UMP^ resistosomes (6, 7). However, conformational changes alone may not drive oligomerization because ETI, while sufficient to induce heterotrimeric interactions, is insufficient for detection of NRG1–EDS1–SAG101 resistosome-like complexes (Fig. 4*B*). Although the assembly of NRG1–EDS1–SAG101 resistosomes could occur below detection thresholds under ETI activation alone, a PTI mechanism clearly promotes its accumulation (Fig. 4*B*). This is in contrast to what is known for ZAR1 (6, 7). Perhaps, PTI-mediated modification(s) of NRG1, EDS1, and/or SAG101 stabilize resistosomes and allow accumulation at the PM to increase cytoplasmic [Ca^2+^] and promote cell death. Alternatively, PTI could more indirectly facilitate the accumulation of NRG1–EDS1–SAG101 resistosomes in vivo: PTI activation leads to transcriptional accumulation of TIR-domain proteins (23) which may enhance EDS1–SAG101-assisted assembly and oligomerization of NRG1 resistosomes through increased production of nucleotide-based small molecules. Our study highlights the importance of future investigations into the possible role of NRG1–EDS1–SAG101 complexes as a mechanistic link for PTI and ETI coactivation of plant defense responses.

## Materials and Methods

### NRG1.1 and NRG1.2.

Arabidopsis carries functionally indistinguishable paralogs NRG1.1 and NRG1.2 (13, 14), as well as the truncated NRG1.3 which is reported to antagonize NRG1-mediated immunity (39). This study employed either NRG1.1 or NRG1.2 to facilitate both biochemistry and cell biology investigations. Previously, it was shown that fusion-tagging NRG1 can interfere with function (12). We generated NRG1.2-GFP stable Arabidopsis lines but these were not functional. To ensure that our investigations were performed with functional tagged alleles, cell biology assays were performed with fluorophore-tagged NRG1.1 variants in both Arabidopsis stable lines and after transient expression in *N. benthamiana*. It was also previously shown that NRG1.2-FLAG and NRG1.1-V5 are functionally indistinguishable in Arabidopsis stable lines (14). We utilized the NRG1.2-FLAG Arabidopsis line to facilitate 3XFLAG peptide elution of native protein states for BN-PAGE assays. Biochemical assays showing an induced association of NRG1.1 or NRG1.2 with EDS1 and SAG101 after transient expression in *N. benthamiana* or in Arabidopsis stable lines, respectively, were reported previously (10).

### Growth of Arabidopsis and *N. benthamiana*.

Arabidopsis plants for pathogen assays were grown with 8 h photoperiod in controlled environment rooms (CERs) at 20 to 22 °C with 70% relative humidity. Arabidopsis plants for seed collection were grown at similar CER conditions and a 16 h photoperiod. *N. benthamiana* plants for transient infiltration and cell death assays were grown with a 16 h photoperiod in a CER at a 20 to 22 °C with 70% relative humidity.

### Preparation of Pf0-1 for Infiltration.

The Effector-to-Host Analyzer (EtHAn) system (24) is a nonpathogenic strain of *P. fluorescens* (Pf0-1) engineered with type III secretion system to deliver encoded effectors into plant cells. Glycerol stocks of Pf0-1 strains carrying effector expression-vectors were incubated 24 to 48 h at 28 °C on King’s B medium with antibiotics as previously described (24). Fresh colonies were cultured and incubated 24 h at 28 °C before suspension in MgCl_2_–MES pH 5.6.

### Pf0-1 Infiltration of Arabidopsis Leaves.

Rosette leaves of Arabidopsis were infiltrated with Pf0-1 strains resuspended at OD_600_ = 0.2 in MgCl_2_–MES pH 5.6. Mock (“MgCl_2_”) infiltrations were MgCl_2_–MES pH 5.6 only. Leaves for protein assays were harvested, flash-frozen in liquid nitrogen, and stored at –80 °C. Plants were ~4-wk-old in cell death complementation assays. Leaves were visualized for macroscopic tissue-collapse 24 h post effector-delivery by Pf0-1 infiltration. Plants were ~4 to 5-wk-old in coIP assays and siblings were visualized for HR 24 h post effector-delivery by Pf0-1 infiltration.

### β-Estradiol Infiltration of Arabidopsis Leaves.

Arabidopsis stable lines carrying the XVE cassette (32) were generated for β-estradiol induction of LexA promoter-driven AvrRps4-mCherry. Leaves of 4 to 5-wk-old Arabidopsis were infiltrated with 50 µM β-estradiol diluted in MgCl_2_–MES pH 5.6 with 0.01% Silwet® L-77. Mock was prepared with dimethyl sulfoxide in place of β-estradiol.

### Agroinfiltration for Cell Death and Coimmunoprecipitation Assays.

Glycerol stocks of *A. tumefaciens* strains were struck on LB agar plates containing antibiotic selections and incubated 48 to 72 h at 28 °C. Cells were collected from plates and resuspended in MgCl_2_–MES pH 5.6 at OD_600_ = 0.1 to 1. Silencing suppressor p19 (40) (OD_600_ = 0.5) and 200 µM acetosyringone were coinfiltrated into leaves of 4 to 6-wk-old *N. benthamiana* leaves. In cell death assays, leaves were spot-infiltrated and evaluated for macroscopic tissue collapse 3 to 7 days post infiltration (dpi). In coIP assays, the entire leaf surface was infiltrated and leaves were harvested 48 to 72 hpi, flash-frozen, and stored at –80 °C.

### Localization and BiFC Analysis with Transgenic Arabidopsis.

Leaves of 4-wk-old transgenic Arabidopsis were infiltrated with (OD_600_ = 0.2) Pf0-1 AvrRps4, Pf0-1 EV, or 10 mM MgCl_2_ (mock) for live cell imaging. Leaf discs were harvested 4 to 6 h post Pf0-1 infiltration and imaged with a Zeiss LSM780 confocal laser scanning microscope for NRG1.1-cCFP and SAG101-nVenus BiFC and with a Zeiss LSM700 confocal laser scanning microscope for NRG1.1-mRuby localization. The following excitation and detection windows were used: YFP 514 nm, 530 to 550 nm, mRuby 560 nm, 607 to 683 nm, autofluorescence of chlorophyll A 550 nm, 695 to 737 nm. Objectives used were 20× (0.8 NA, water), 40× (1.3 NA oil). DAPI staining was used to mark nuclei. Confocal images were compiled using ImageJ and Z-stacks were projected with Fiji using SD methods.

### Localization and BiFC Analysis with *N. benthamiana* epss Reconstitution Assays.

*Agrobacterium* carrying BiFC constructs was syringe-infiltrated into leaves of *N. benthamiana* lacking all EDS1-family proteins (*Nb*_*epss*) (12) for live cell imaging. At 48 hpi of *Agrobacterium*, the leaf zone was subsequently infiltrated with (OD_600_ = 0.3) Pf0-1 XopQ, Pf01 EV, or 10 mM MgCl_2_ (mock). At 4 to 6 hpi of Pf0-1, leaf discs were harvested for confocal microscopy imaging. A Leica TCS SP8 confocal laser scanning microscope was used. Excitation and detection windows used: YFP 514 nm, 530 to 550 nm, autofluorescence of chlorophyll A 550 nm, 695 to 737 nm. Objectives used: 20× (0.8 NA, water), 40× (1.3 NA oil or 1.2 water). Confocal images were compiled using ImageJ, and Z-stacks were projected with Fiji using SD methods.

### Leaf Protein Accumulation Assays.

Leaf protein accumulation assays in *SI Appendix,* Fig. S3 were performed as described in ref. 27.

### Arabidopsis Lysate Preparations.

Protein was purified from ~2.5 to 3.5 g dry-weight Arabidopsis leaves flash-frozen in liquid nitrogen. Tissue was ground by mortar and pestle in liquid nitrogen and membranes were solubilized in 100 mM HEPES (pH 7.5), 300 mM NaCl, 5 mM MgCl_2_, 0.5% Nonidet™ P-40 Substitute (11754599001), 10% glycerol, 2% polyvinylpolypyrrolidone, 1 tablet complete™, ethylenediaminetetraacetic acid (EDTA)-free protease inhibitor cocktail tablet, 10 mM DTT. Lysates were incubated inverting 10 min at 4 °C before centrifugation at 4 °C 35 min at 4,000 × g. Lysates were filtered through Miracloth (475855). Input samples were combined with sodium dodecyl sulphate (SDS) sample buffer and 10 mM DTT and heated at 65 °C for 5 min before storage at –20 °C.

### Coimmunoprecipitation with Arabidopsis Lysates.

Approx. 3 to 4 mL soluble lysate was combined with 50 µL Anti-FLAG^®^ M2 Affinity Gel slurry and inverted ~2 h at 4 °C. Samples were centrifuged at 800 × g for 5 min at 4 °C, the supernatant was removed, and beads were washed [100 mM HEPES (pH 7.5), 300 mM NaCl, 5 mM MgCl_2_, 0.5% Nonidet™ P-40 Substitute, 10% glycerol] inverting at 4 °C for 5 min for a total of three washes. Elution was performed in 100 µL wash buffer with 0.5 mg/mL 3×FLAG^®^ peptide. The sample was incubated ~2.5 h at 4 °C shaking at 750 RPM for 5 min every 25 min. The elution product was transferred to fresh 1.5 mL Eppendorf. Beads were heated at 65 °C for 5 min in SDS sample buffer and 100 mM DTT. The elution product was combined with SDS sample buffer and 10 mM DTT, heated 65 °C for 5 min, and stored at –20 °C.

### *N. benthamiana* Lysate Preparations.

As described for Arabidopsis except that ~0.5 to 1 g dry-weight leaf tissue was used.

### Coimmunoprecipitation with *N. benthamiana* Lysates.

As described for Arabidopsis except that 1.5 mL lysate was combined with 20 µL Anti-FLAG^®^ M2 Affinity Gel slurry.

### Golden Gate Assembly of DNA Constructs.

Golden Gate cloning (41) was used for the generation of stable Arabidopsis lines and transient expression in *N. benthamiana*. Arabidopsis lines for coIP were generated with native promoters and terminators with the exception of pSAG101:SAG101-HA:HSP18t. Arabidopsis lines for BiFC were generated with native promoters and terminators with the exception of pSAG101:SAG101-nVenus:35St. Transient expression constructs for coIP were generated with 35S promoters and Ocs terminators. Transient expression constructs for BiFC were generated with 35S promoters and 35S terminators and NRG1.1, NRG1.1^EEAA^, EDS1, EDS1^H476Y^, and SAG101 genomic sequences as described previously (10). C-terminal cCFP and nVenus tags were described previously (42). “FLAG” tag refers to 6×HIS-3×FLAG^®^ and “HA” refers to 6×HA. Golden Gate compatible epitope tag mRuby was synthesized by Thermo Fisher Scientific.

### SDS-PAGE.

SDS sample buffer was prepared to a 4× concentration at 250 mM Tris-HCl (pH 6.8), 8% (w/v) sodium dodecyl sulphate (SDS), 40% glycerol (v/v), 50 mM EDTA, and bromophenol blue for visualization. The samples were prepared with SDS sample buffer to a final concentration of 1×, incubated 5 min at 65 °C, and stored at –20 °C. Frozen samples were warmed to 37 °C for 5 min, briefly vortexed, and centrifuged at 15,000 × g for 1 min. Samples were resolved by SDS-PADE (precast 4 to 20% Mini-PROTEAN^®^ TGX™: 4561095) assembled in gel tank apparatus (Mini-PROTEAN^®^ system) with 1× SDS buffer [25 mM Tris, 200 mM Glycine, 2% SDS (w/v)]. Samples were loaded alongside 5 µL PageRuler™ Prestained Protein Ladder 10 to 180 kDa (26617). Electrophoresis was run at 90 V for ~15 min then 135 V for ~45 min or until dyeedge migrated to gel base. Input membranes were ponceau-stained as loading control.

### Blue Native PAGE.

BN-PAGE was performed according to the NativePAGE™ Novex^®^ Bis-Tris Gel System with precast NativePAGE™ 3 to 12% Bis-Tris Mini Protein Gels (10-well BN1001BOX) and 5 µL marker (see below). Electrophoresis was run at 150 V for ~60 min then at 250 V for ~45 min until dye-edge migrated to gel base. Electrophoresis was run with dark cathode buffer for ~25 min and then with light cathode buffer the remainder of the run. The gel was subjected to semidry transfer (described below). The membrane was incubated for 15 min in 8% acetic acid, rinsed, and air-dried at room temperature (RT) >1 h. The membrane to detect NRG1.2-V5 lysate and α-FLAG elution product was incubated overnight in 1X SDS buffer at RT rotating 60 RPM. Markers were visualized when the membrane was preactivated with ethanol. Figures were generated with SDS-PAGE and western blot analysis of an identical sample.

### Blue Native PAGE Markers.

BN-PAGE gels were loaded with 5 µL of either NativeMark™ Unstained Protein Standard (LC0725), or SERVA Native Marker (39219.01). Migrations are not consistent between figures and this correlates with arrival of a different stock of NativeMark™. Migrations in Figs. 2*C* and 3*A* are consistent and were run with the previous stock of NativeMark™, while those in Fig. 4*B* and *SI Appendix*, Figs. S6 and S7 *A* and *C* were run with the new stock of NativeMark™ and show a different but consistent migration pattern. Fig. 3*B* and *SI Appendix*, Fig. S7*B* were run with SERVA and show a consistent migration pattern.

### Semidry Protein Transfer and Western Blotting.

Semidry transfer was performed using Bio-Rad Trans-Blot® Turbo™ Transfer System (1620177; Midi 0.2 µM polyvinylidene difluoride (PVDF) Transfer Kit: 1704273) in standard settings. PVDF membranes were blocked 30 to 60 min, rotating 60 RPM at RT, with 5% milk TBS-T (50 mM Tris-HCl pH 8.0, 150 mM NaCl, 0.1% Tween^®^-20) before immunolabelling with α-V5-horseradish peroxidase (HRP), α-HA-HRP, α-FLAG^®^-HRP, or α-Myc-HRP in 5% milk TBS-T overnight at 4 °C or >1 h at RT rotating 60 RPM. AvrRps4-mCherry and NRG1.1-mRuby were immunolabelled with α-mCherry, washed, and reprobed with α-rabbit (Rb)-HRP. Membranes were washed three times for 5 to 10 min with TBS-T then three times for 5 to 10 min with TBS rotating RT at 60 RPM. Membranes were incubated with SuperSignal™ West Pico PLUS (34580) or West Femto (34095) Chemiluminescent Substrate and imaged with GE Healthcare ImageQuant™ LAS 4000 or Amersham ImageQuant™ 800 enhanced chemiluminescence systems.

## Supplementary Material

Appendix 01 (PDF)

## Data Availability

All study data are included in the article and/or *SI Appendix*.
