# Supplementary material for: Oligomerization of a plant helper NLR requires cell-surface and intracellular immune receptor activation
Source: Proc Natl Acad Sci U S A. 2023 Mar 6;120(11):e2210406120. doi: 10.1073/pnas.2210406120 (PMC10089156; doi:10.1073/pnas.2210406120)
Supplement: Supplementary file 1 — Appendix 01 (PDF) [file pnas.2210406120.sapp.pdf]

Supplementary Figures

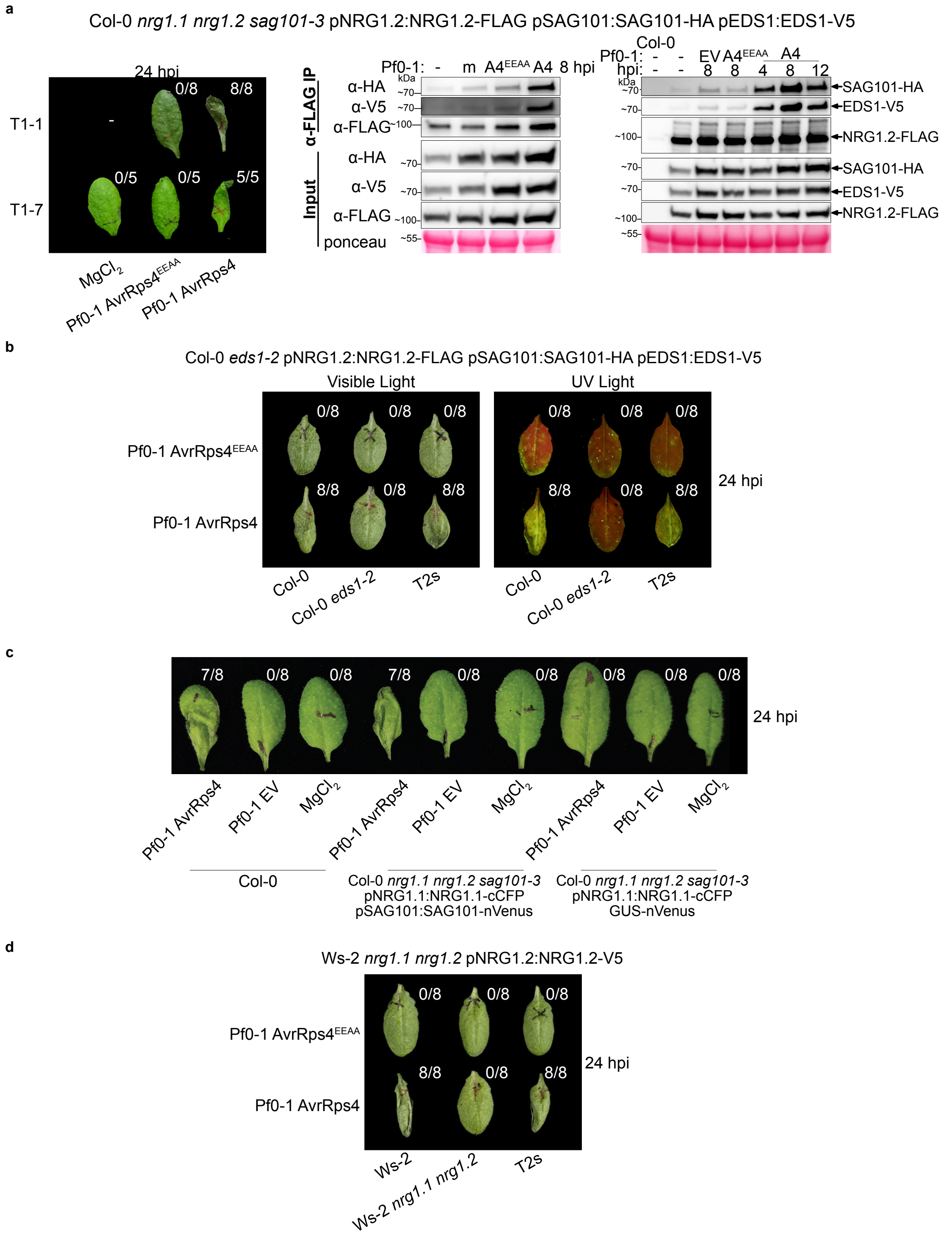

Fig. S1. Cell death complementation in Arabidopsis stable transgenic lines. (a-d) Numbers in white indicate number of leaves showing visual tissue collapse over total number of infiltrated leaves per genotype. Images were collected 24 hpi and a representative biological replicate is shown. **(a, left)** Stable expression of pNRG1.2:NRG1.2-FLAG and pSAG101:SAG101-HA complement Col-0 *nrg1.1 nrg1.2 sag101-3*. Pf0-1 AvrRps4 induces macroscopic cell death in leaves of two

independent Arabidopsis lines. Pf0-1 AvrRps4^{EEAA} and $MgCl_2$ are used as negative controls. Similar results were observed in more than three independent biological replicates. Col-0 control represented in (c). **(a, middle)** NRG1 weakly associates with EDS1 and SAG101 upon cell-surface receptor activation in Arabidopsis. Experiments were performed on three biological replicates each for two independent lines, for a total of six replicates, with similar results. SDS-PAGE and Western blots of coIPs performed with native promoter-driven Arabidopsis stable line. "A4" indicates AvrRps4, "A4^{EEAA}" indicates AvrRps4^{EEAA}, "m" indicates $MgCl_2$ mock, and "-" indicates un-infiltrated. **(a, right)** Effector delivery in Arabidopsis induces NRG1 interaction with EDS1 and SAG101. Experiments were performed on three biological replicates with similar results. SDS-PAGE and Western blots of coIPs performed with native promoter-driven Arabidopsis stable line. **(b)** Stable expression of pEDS1:EDS1-V5 complements Col-0 *eds1-2*. This was performed to confirm functionality of pEDS1:EDS1-V5 in (a) lines. Pf0-1 AvrRps4 induces macroscopic cell death in leaves of eight independent T2 generation individuals. Col-0 is used as a positive control and Col-0 *eds1-2* is used as a negative control for AvrRps4 recognition. Pf0-1 AvrRps4^{EEAA} is used as a negative control for cell death. White light images were collected to demonstrate tissue collapse. Ultraviolet light images were collected to demonstrate cell leakage. **(c)** Stable expression of pNRG1.1:NRG1.1-cCFP and pSAG101:SAG101-nVenus complement Col-0 *nrg1.1 nrg1.2 sag101-3* while 35S:GUS-nVenus does not. Col-0 was used as a positive control for AvrRps4 recognition. Pf0-1 EV and $MgCl_2$ were used as negative controls for cell death. Three biological replicates were performed in one stable line with similar results. **(d)** Stable expression of pNRG1.2:NRG1.2-V5 complements Ws-2 *nrg1.1 nrg1.2*. This was performed to confirm functionality of pNRG1.2:NRG1.2-V5 in super-transformed lines (Fig. 2). Pf0-1 AvrRps4 induces macroscopic cell death in leaves of eight independent T2 generation individuals. Ws-2 was used as a positive control and Ws-2 *nrg1.1 nrg1.2* was used as a negative control for AvrRps4 recognition. Pf0-1 AvrRps4^{EEAA} is used as a negative control for cell death.

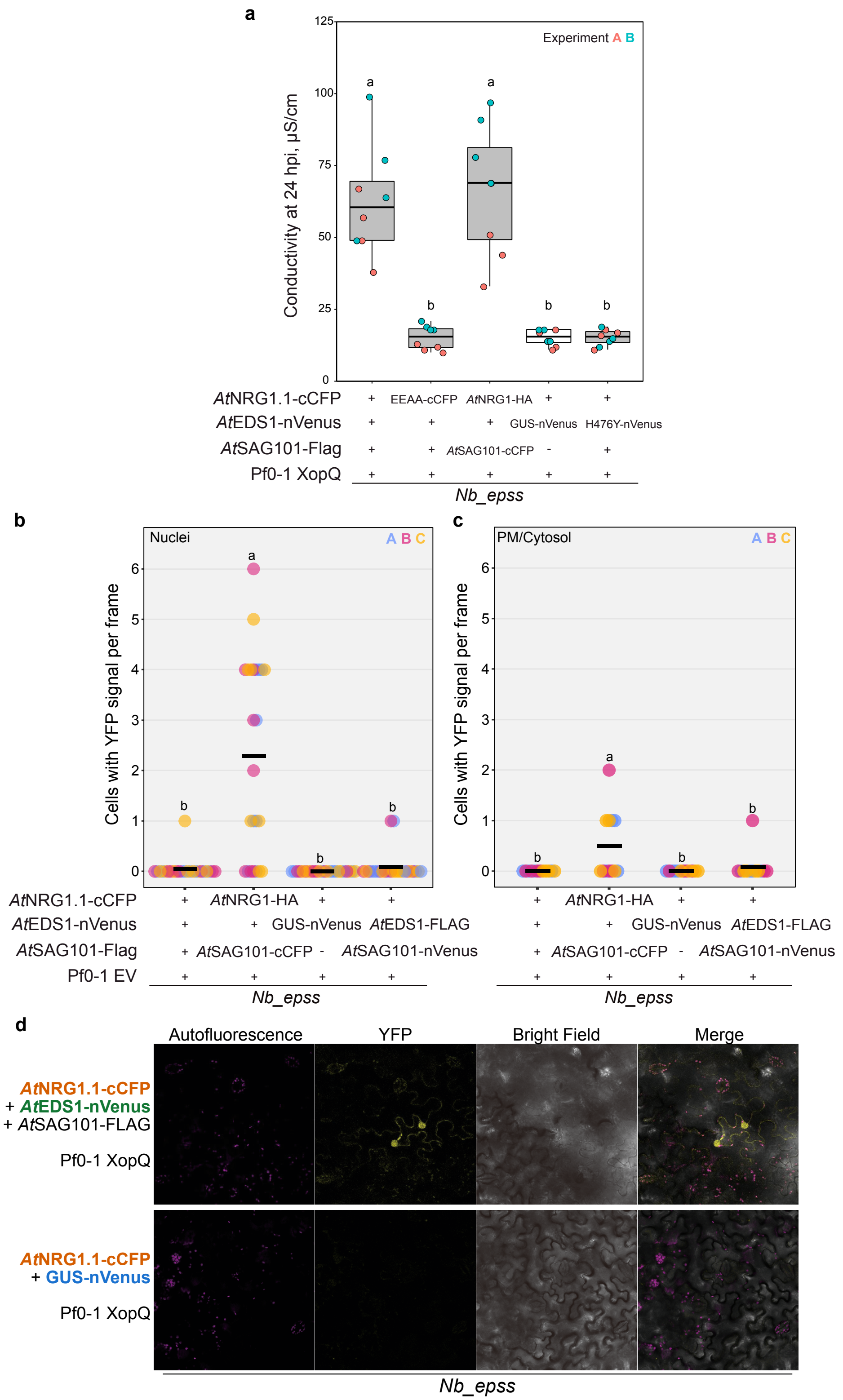

Fig. S2. NRG1-EDS1 or NRG1-SAG101 BiFC signal not detectable in absence of effector. **(a)** Constructs used in Fig. 1b-d and Fig. S2b-c restore XopQ-triggered cell death in *Nb_epss* mutant background. Cell death was quantified in electrolyte leakage assays 6 h after harvesting

leaf discs (24 hpi of Pf0-1 XopQ). Leaves were Agro-infiltrated 48 h prior to Pf0-1 infiltration with expression vectors carrying 35S promoter-driven *At*NRG1.1-cCFP or *At*NRG1.1^{EEAA}-cCFP with *At*EDS1-nVenus, AtEDS1^{H476Y}-nVenus, or GUS-nVenus, and *At*SAG101-FLAG. Experiments were performed two times independently, each with four replicates (leaf discs) (Tukey HSD, α=0.001, n=8). Error bars represent standard error of mean. Letters indicate significantly different groups. Datapoints with the same color come from one independent experiment. **(b)** NRG1-EDS1 or NRG1-SAG101 BiFC signal is not detectable at PM/cytosol in the absence of effector. Numbers of cells with BiFC-mediated YFP signal in PM/cytoplasm in *N. benthamiana* leaves were counted. Experiments were performed three times independently, each with four leaf-disc replicates (n=12). Mean indicated at black line. ANOVA, $P < 0.001$, Tukey HSD, $P < 0.05$. **(c)** NRG1-EDS1 or NRG1-SAG101 BiFC signal is not detectable in nuclei in the absence of effector. Numbers of cells with BiFC-mediated YFP signal in the nucleus in *N. benthamiana* leaves were counted. Experiments were performed three times independently, each with four leaf-disc replicates (n=12). Mean indicated at black line. ANOVA, $P < 0.001$, Tukey HSD, $P < 0.05$. Data in (a) (b) and (c) generated with ggplot2 (3.3.2) package in R. **(d)** NRG1 associates with EDS1 and not GUS in nuclei and PM/cytoplasm in activated *N. benthamiana leaves*. *Agrobacteria* carrying 35S promoter-driven BiFC constructs were infiltrated into *Nb_epss* leaves. At 48 hpi, Pf0-1 was infiltrated. At 4-6 hpi, leaf disks were imaged. Representative images are shown for three independent biological replicates, each with four technical replicates, with similar results. Autofluorescence (magenta) and YFP (yellow) signal are shown.

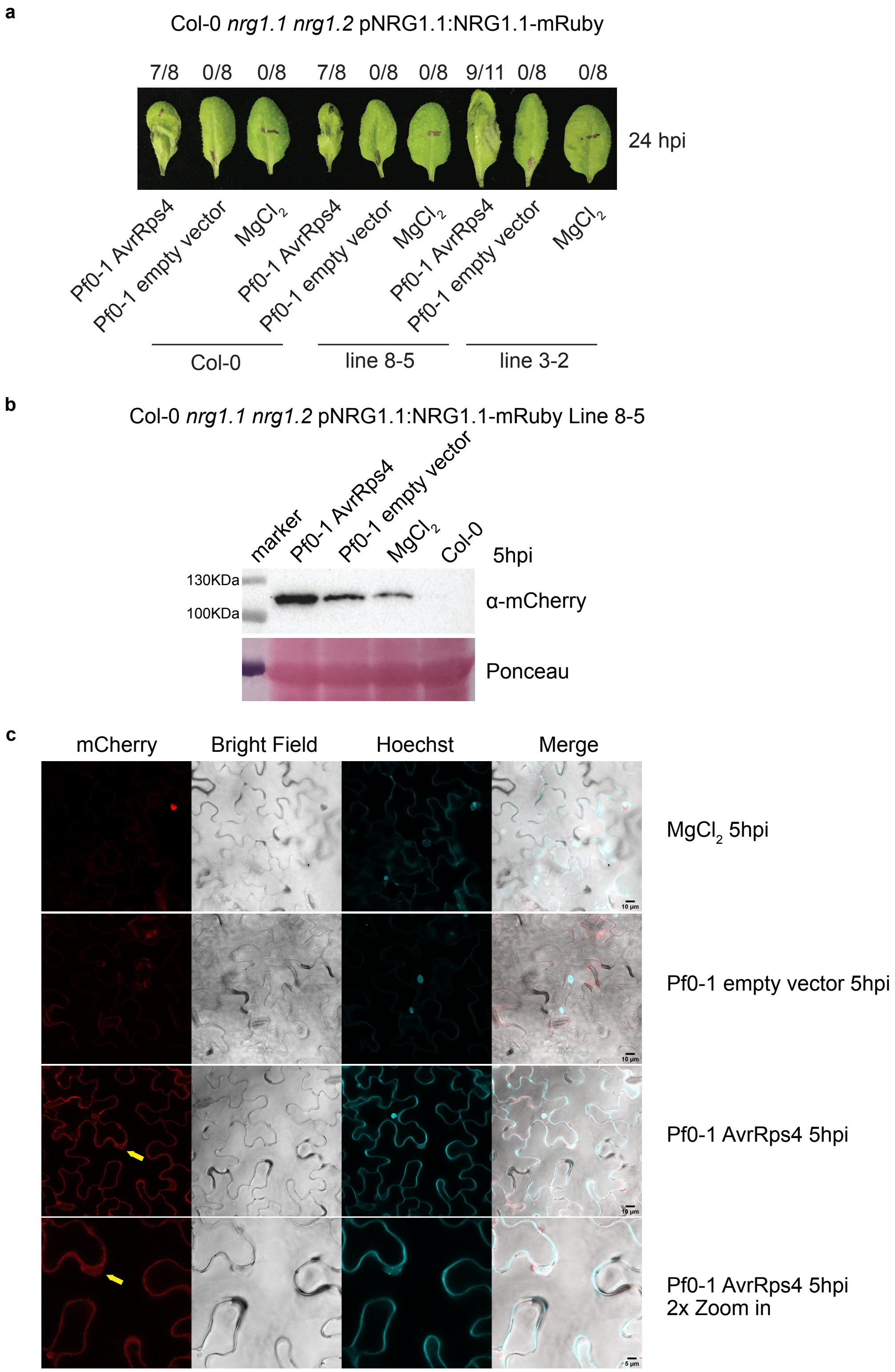

Fig. S3. NRG1-mRuby is localized to nuclei upon AvrRps4 delivery by Pf0-1. **(a)** Macroscopic cell death in leaves of two independent lines of Col-0 *nrg1.1 nrg1.2* carrying pNRG1.1:NRG1.1-mRuby 24 hpi of Pf0-1 AvrRps4. Wild type Col-0 was used as positive control. Infiltration of Pf0-1 EV or

$MgCl_2$ served as negative controls. Numbers indicate leaves showing visual tissue collapse over total infiltrated leaves per genotype. Data was collected in three independent experiments. Representative images of one biological replicate are shown. **(b)** Higher accumulation of NRG1.1 is detected upon effector delivery in Arabidopsis. Leaves Col-0 *nrg1.1 nrg1.2* carrying pNRG1.1:NRG1.1-mRuby were infiltrated with Pf0-1 AvrRps4, Pf0-1 EV, or $MgCl_2$. In three independent replicates, four leaf discs were harvested at 5 hpi for SDS-PAGE and Western blot analyses of NRG1.1 protein accumulation. **(c)** NRG1.1 is detected at the PM/cytoplasm and in nuclei upon effector delivery in Arabidopsis. Live cell imaging of pNRG1.1:NRG1.1-mRuby stably expressed in Col-0 *nrg1.1 nrg1.2*. All images show single planes and micrographs were taken 4-5 hpi. Localization was determined in three independent experiments and representative micrographs are shown. PM/cytoplasm signal was detected in every cell and 2-4 nuclei could be imaged in one frame. Hoechst (cyan) and mCherry (red) signal are shown.

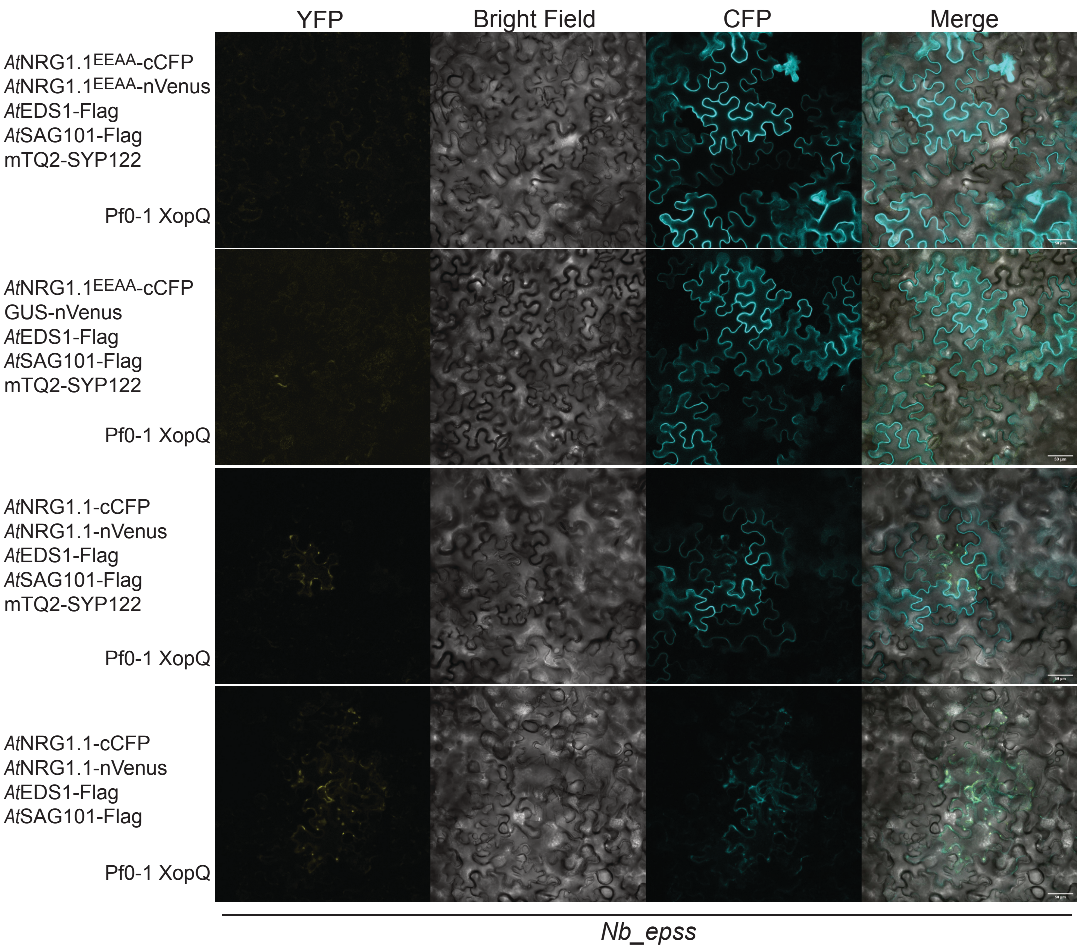

Fig. S4. NRG1-NRG1 self-association is undetectable by BiFC. *Agrobacteria* carrying 35S promoter-driven BiFC constructs were infiltrated into *Nb_epss* leaves. At 48 hpi, Pf0-1 XopQ was infiltrated. At 8 hpi, leaf disks were imaged. Three independent biological replicates, each with four technical replicates, were performed with similar results and representative images are shown. mTQ2-SYP122 served as PM marker. Scale bar = 50 μm.

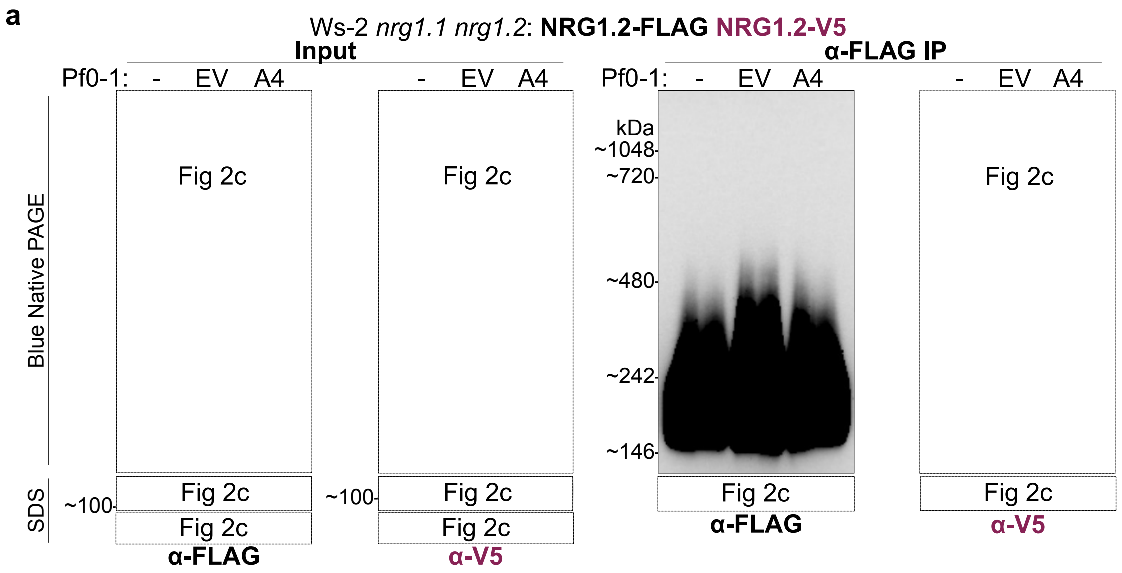

Fig. S5. Overexposure does not indicate slower migrating species of NRG1.2-FLAG after α-FLAG IP. **(a)** BN-PAGE and Western blot of elution products from native promoter-driven stable Arabidopsis lines. Immunoblot is overexposure of blot shown in the same position in Fig. 2c.

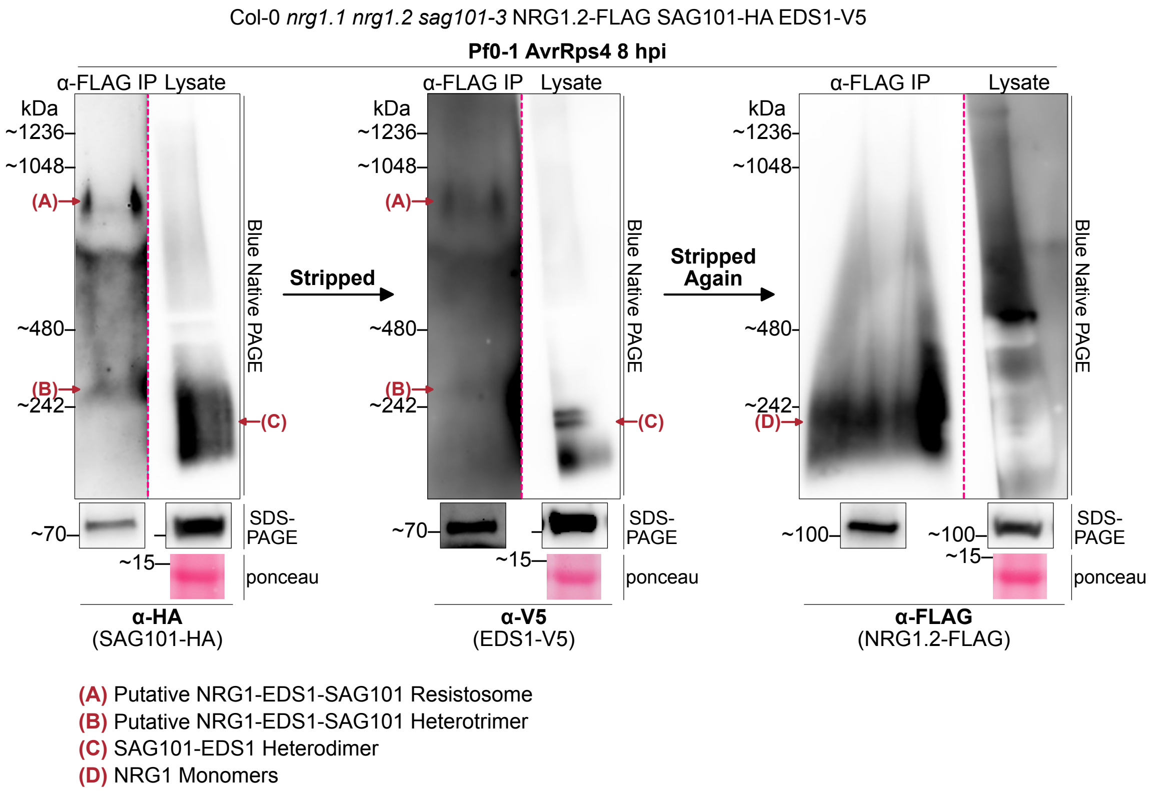

Fig. S6. Faster migrating species of SAG101-HA and EDS1-V5 after α-FLAG IP of NRG1.2-FLAG in immune-activated tissues migrate slower than pre-activated EDS1-SAG101 heterodimers and NRG1.2 monomers. BN-PAGE and Western blot of lysates and coIP elution products from native promoter-driven stable Arabidopsis lines. The same membrane is shown after sequential immunolabelling and stripping with Restore^{TM} Western Blot Stripping Buffer (21059). First immunolabelling was α-HA to detect SAG101-HA, second immunolabelling was α-V5 to detect EDS1-V5, and third immunolabelling was α-FLAG to detect NRG1.2-FLAG. Left lane shows elution product after α-FLAG IP of NRG1.2-FLAG while right lane shows lysate. Left lane is representative of post-activation complex formations for EDS1 and SAG101, and right lane is representative of pre-activation states for NRG1, EDS1, and SAG101 which do not change in lysates from mock/un-infiltrated to AvrRps4-treated (Fig. 3). Lysate in α-FLAG blot shows bleaching effects, likely an artifact of sequential stripping. In α-FLAG blot, α-FLAG IP shows pre-activation state of NRG1.2-FLAG as large NRG1 oligomer is not observed, and banding patterns do not change, from mock/un-infiltrated to AvrRps4-treated and observation of oligomeric NRG1 requires detection of NRG1.2-V5 after α-FLAG IP of NRG1.2-FLAG (Fig. 3a & 3c). Species identities are indicated in red, and red dashed lines indicate lanes were cropped. Samples were run in parallel on same membrane. Two independent biological replicates showed similar results.

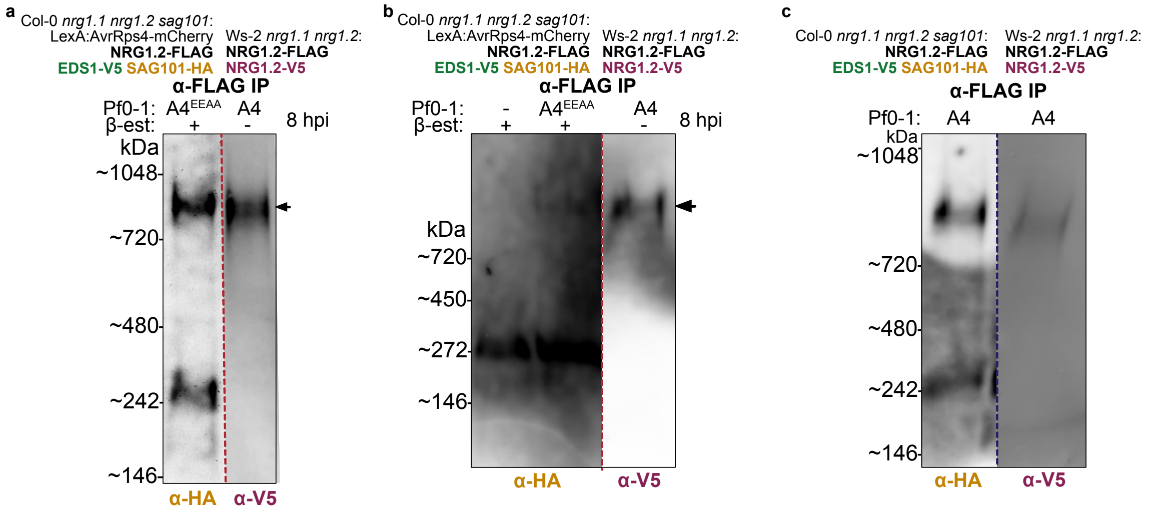

Fig. S7. Biological replicates show co-migration of NRG1-EDS1-SAG101 putative resistosome. Biological replicates for Fig. 3b showing comigration of high molecular weight species of SAG101-HA and NRG1.2-V5 after α-FLAG IP. BN-PAGE of coIP elution products performed with native promoter-driven Arabidopsis stable lines. **(a)** Second biological replicate. The α-HA blot is a duplicate of that in Fig. 4b. NativeMark™ Unstained Protein Standard was used. **(b)** Third biological replicate. SERVA Native Marker was used. **(c)** Fourth biological replicate. NativeMark™ Unstained Protein Standard was used. Red dotted line in (a) and (b) indicates samples were resolved side-by-side on the same gel, the membrane was cut prior to immunolabelling, and the Western blot images were cropped together. Blue dotted line in (c) indicates samples were resolved on the same gel, with other samples in between, and the Western blot images were cropped together.

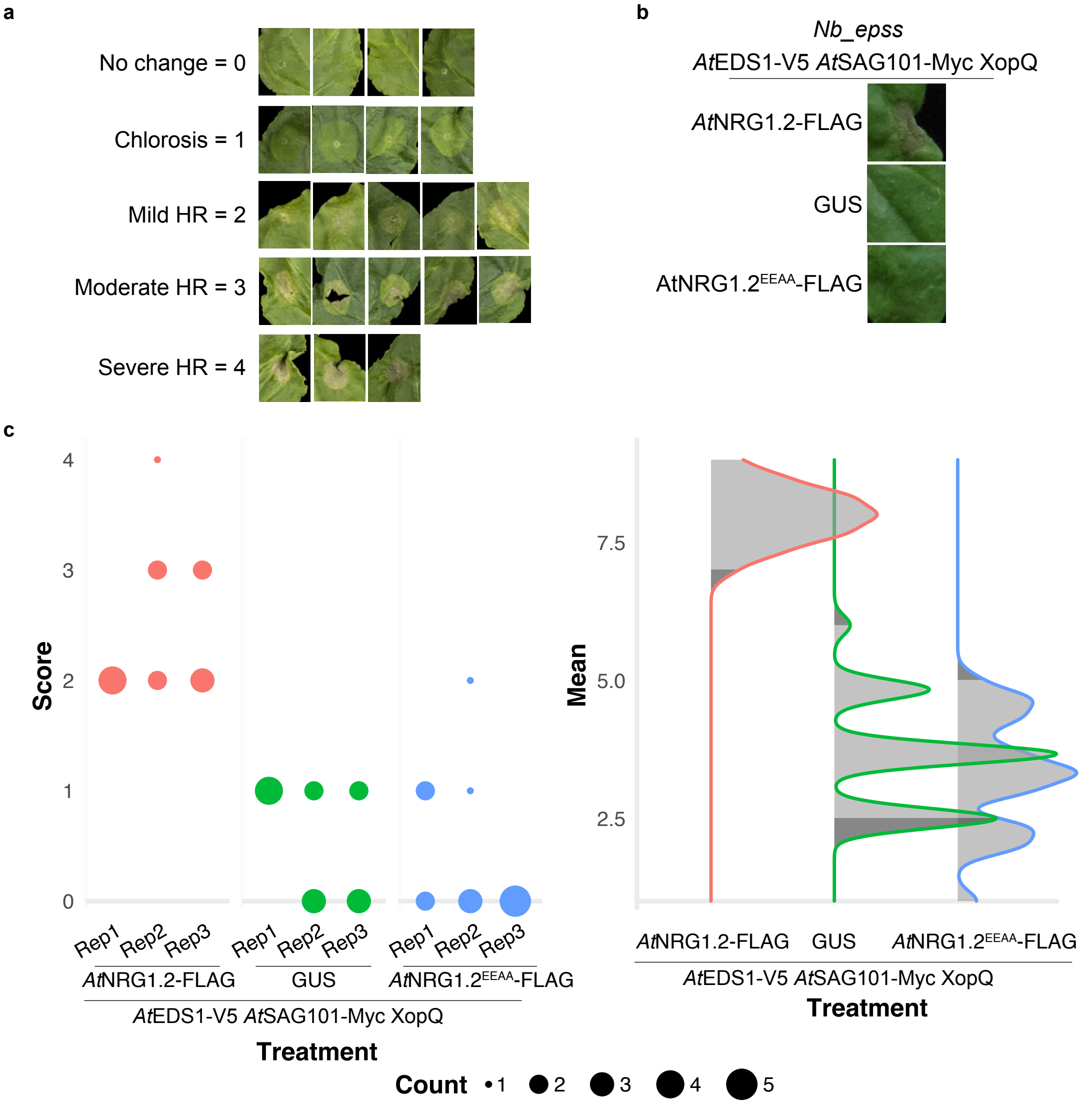

Fig. S8. *At*NRG1.2^{EEAA}-FLAG is defective in cell death assays in *N. benthamiana*. These data replicate what was observed for *At*NRG1.1^{EEAA} (1). **(a)** A bespoke cell death scoring system was generated for Agro-infiltration mediated reconstitution of Arabidopsis NRG1, EDS1, SAG101 in *Nb_epss* (2). A score of 0 (no change) and 1 (chlorosis) were not considered cell death. Representative images are shown from cell death assays in more than three representatives. **(b)** Representative images of one biological replicate from (c). Agro-mediated expression of 35S promoter-driven *At*EDS1-V5, *At*SAG101-Myc, and XopQ with *At*NRG1.2-FLAG, *At*NRG1^{EEAA}-FLAG or GUS in *Nb_epss*. Cell death is visualized as tissue collapse and was scored 7 days post Agro-infiltration. **(c)** Replacement of glutamate residues with alanine in the N-termini of *At*NRG1.2^{EEAA} -FLAG results in loss of cell death phenotypes. Results are visualized in Derevnina plots (3). Dot plot size is proportional to sample number with confidence interval peaks adjacent. Experiment was performed with three independent biological replicates, each with 3-6 technical replicates. Estimation statistical tests were implemented with besthr R library (4). Bootstrapping resampling tests were performed with lower significance cut-off of 0.025 and upper of 0.975. Non-overlapping confidence interval peaks were considered significant. Data generated with ggplot2 (3.3.2) package in R.

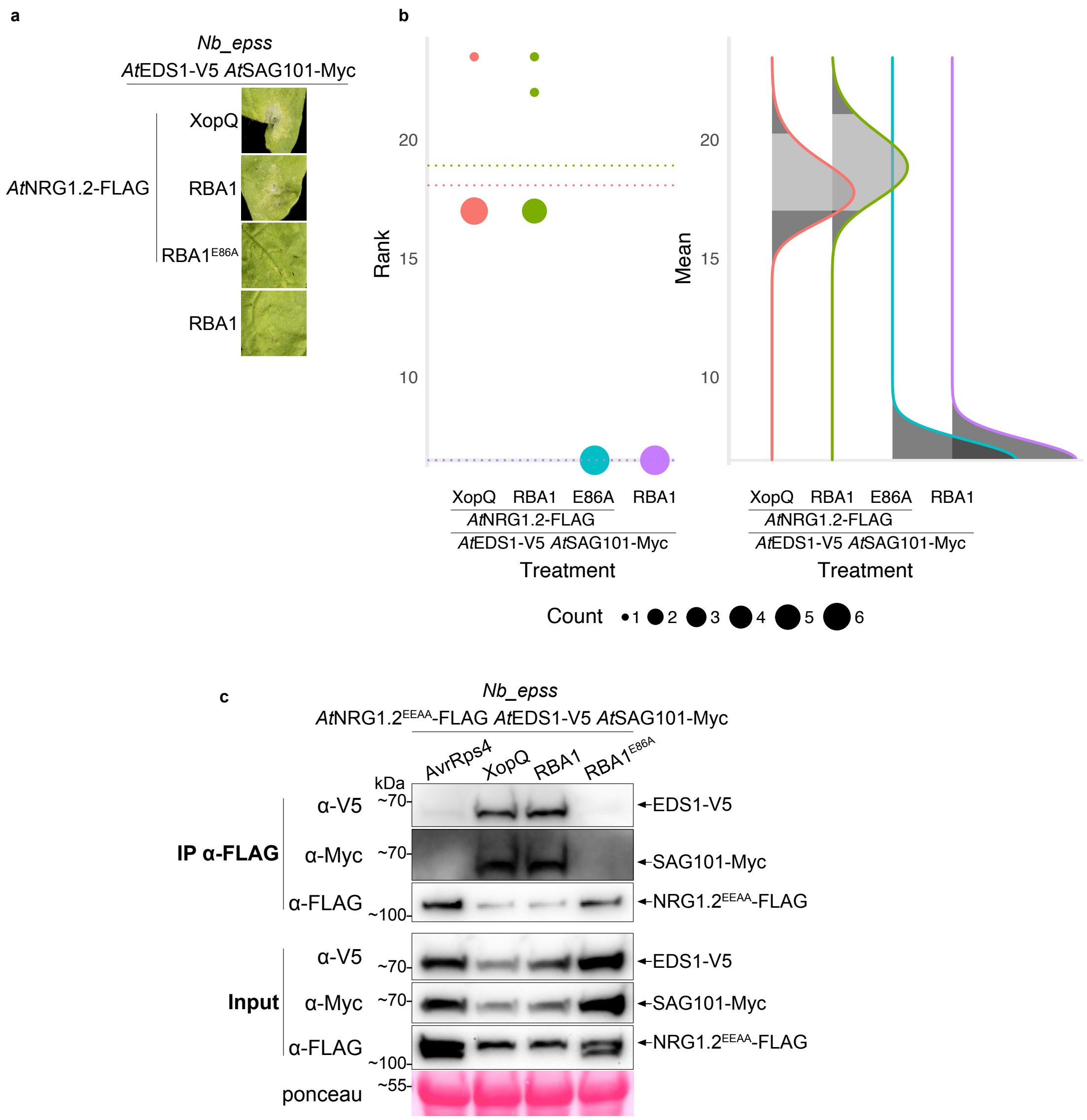

Fig. S9. RBA1 induces EDS1 and SAG101 association with NRG1, which is lost when co-expressed with RBA1^{E86A}. **(a)** Representative images of one biological replicate from (b). Agro-mediated expression of 35S promoter-driven *At*EDS1-V5 and *At*SAG101-Myc, with or without *At*NRG1.2-FLAG, and XopQ, RBA1, or RBA1^{E86A} in *Nb_epss*. Cell death is visualized as tissue collapse and was scored 7 days post Agro-infiltration. **(b)** Replacement of glutamate residue with alanine in RBA1^{E86A} does not induce cell death in Arabidopsis NRG1, EDS1, SAG101 reconstitution assays (2, 5). Results are visualized in Derevnina plots (3) with dot plot size proportional to sample number and with adjacent confidence interval peaks. Estimation statistical tests were implemented with besthr R library (4). Bootstrapping resampling tests were performed with lower significance cut-off of 0.025 and upper of 0.975. Non-overlapping confidence interval peaks were considered significant. **(c)** Replacement of glutamate residue with alanine in RBA1^{E86A} abolishes the RBA1-induced association of EDS1 and SAG101 with NRG1.2^{EEAA}. SDS-PAGE and Western blot of coIP after Agro-infiltration and transient expression of 35S promoter-driven *At*NRG1^{EEAA}-FLAG, *At*EDS1-V5, and *At*SAG101-Myc with AvrRps4, XopQ, RBA1, or RBA1^{E86A} in *Nb_epss*. NRG1.2^{EEAA} was

used to remove confounding variable of cell death. Tissue was harvested 48 hpi. Co-delivery of XopQ was utilized to activate TNL signaling and induce *At*NRG1.2^{EEAA}-FLAG association with AtEDS1-V5 and *At*SAG101-Myc. AvrRps4 was used as a negative control for TNL activation in *N. benthamiana*. Experiments were performed on three independent biological replicates with similar results. Data generated with ggplot2 (3.3.2) package in R.

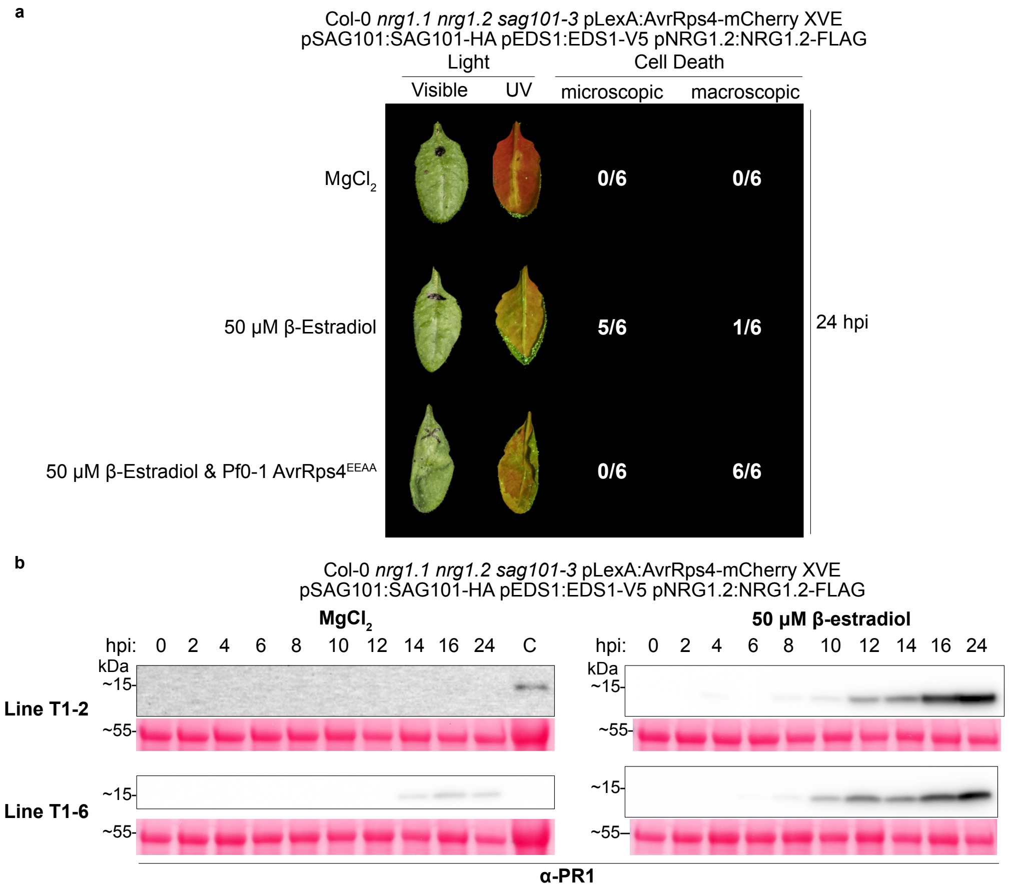

Fig. S10. β-estradiol treatment in inducible lines reproduces microscopic cell death and defense gene activation. **(a)** β-estradiol-mediated induction of AvrRps4-mCherry with XVE system induces microscopic cell death, and β-estradiol co-infiltration with Pf0-1 $AvrRps4^{EEAA}$ reconstitutes macroscopic cell death in stated Arabidopsis stable transgenic line (6). $MgCl_2$ is used as a negative control. White light images were collected to demonstrate tissue collapse. Ultraviolet light images were collected to demonstrate autofluorescent cell leakage. Numbers in white indicate number of leaves showing autofluorescence (microscopic) or visual tissue collapse (macroscopic)/total number of infiltrated leaves per genotype. Images were collected 24 hpi and a representative of one biological replicate is shown. Experiment was performed in two independent Arabidopsis stable lines to reconfirm Ngou et al. 2020 (6). **(b)** β-estradiol-mediated induction of AvrRps4-mCherry with XVE system induces defense activation (7). SDS-PAGE and Western blot of lysates for leaf protein accumulation assays. Defense activation was evaluated by immunolabelling for native α-PR-1 (Agrisera: AS10 687). $MgCl_2$ was used as negative control. Leaves were painted to control for damage-induced PR-1 induction. "C" indicates positive control for α-PR-1 detection. Experiment was performed in two independent Arabidopsis stable lines to reconfirm Ngou et al. 2021 (7).

Supplementary Figure References

1. X. Sun, *et al.*, Pathogen effector recognition-dependent association of NRG1 with EDS1 and SAG101 in TNL receptor immunity. *Nat. Commun.* **12**, 3335 (2021).
2. D. Lapin, *et al.*, A Coevolved EDS1-SAG101-NRG1 Module Mediates Cell Death Signaling by TIR-Domain Immune Receptors. *Plant Cell* **31**, 2430–2455 (2019).
3. L. Derevnina, *et al.*, Plant pathogens convergently evolved to counteract redundant nodes of an NLR immune receptor network. *PLoS Biol.* **19**, e3001136 (2021).
4. D. MacLean, *TeamMacLean/besthr: Initial Release* (2019) https:/doi.org/10.5281/zenodo.3374507.
5. L. Wan, *et al.*, TIR domains of plant immune receptors are NAD+-cleaving enzymes that promote cell death. *Science* **365**, 799–803 (2019).
6. B. P. M. Ngou, *et al.*, Estradiol-inducible AvrRps4 expression reveals distinct properties of TIR-NLR-mediated effector-triggered immunity. *J. Exp. Bot.* **71**, 2186–2197 (2020).
7. B. P. M. Ngou, H.-K. Ahn, P. Ding, J. D. G. Jones, Mutual potentiation of plant immunity by cell-surface and intracellular receptors. *Nature* **592**, 110–115 (2021).
